# Taxonomic synopsis of invasive and native *Spartina* (Poaceae, Chloridoideae) in the Pacific Northwest (British Columbia, Washington and Oregon), including the first report of *Spartina ×townsendii* for British Columbia, Canada

**DOI:** 10.3897/phytokeys.10.2734

**Published:** 2012-03-21

**Authors:** Jeffery M. Saarela

**Affiliations:** 1 Research & Collections, Canadian Museum of Nature, P.O. Box 3443 Stn. D, Ottawa, Ontario K1P 6P4, Canada

**Keywords:** invasive grasses, voucher specimens, coastal habitats, *Spartina*, grass taxonomy, species identification

## Abstract

Five species of the grass genus *Spartina* are invading salt marshes along the Pacific coast of North America, of which three have been documented in British Columbia, Canada, in only the last decade. A taxonomic synopsis of the two native (*Spartina gracilis*, *Spartina pectinata*) and five introduced *Spartina* taxa (*Spartina anglica*, *Spartina alterniflora*, *Spartina densiflora*, *Spartina patens*, *Spartina ×townsendii*) in the Pacific Northwest is presented to facilitate their identification, including nomenclature, a new taxonomic key, new descriptions for a subset of taxa, and representative specimens. *Spartina ×townsendii* is newly reported for the flora of British Columbia. The non-coastal species *Spartina pectinata* is reported from an urban site in British Columbia, the first confirmed report of the taxon for the province. Lectotypes are newly designated for *Spartina anglica* C.E. Hubb., *Spartina maritima* subvar. *fallax* St.-Yves, and *Spartina cynosuroides* f. *major* St.-Yves.

## Introduction

*Spartina* Schreb. (cordgrass) is a small grass genus of some fifteen species native to North America, South America, and the Atlantic coasts of Europe and Africa occurring in such coastal habitats as intertidal mud flats, estuaries, salt marshes, and inland in marshes, sloughs and dry prairie. *Spartina* includes several globally invasive species (e.g., *Spartina alterniflora* Loisel., *Spartina anglica* C.E. Hubb., *Spartina densiflora* Brongn.) that are rapidly altering salt marsh and estuary ecosystems (e.g., Grosholz et al. 2009, Strong and Ayres 2009) and is an important model system for studying allopolyploid speciation and the relationship between hybridization, polyploidy, and invasion (Ainouche et al. 2004a, 2004b, 2009, Baumel et al. 2002a, Salmon et al. 2005, Fortune et al. 2007, 2008, Parisod et al. 2009, Chelaifa et al. 2010). Consequently, a large and broad body of biological, genetic, and ecological knowledge on cordgrasses has been generated. Nevertheless, identification of *Spartina* species can be challenging, as several are morphologically similar and rapidly expanding their ranges into new areas in which up-to-date taxonomic resources for the genus are not available. This is the situation in the Pacific Northwest of North America (Oregon, Washington, British Columbia), where five *Spartina* species are invading coastal areas (*Spartina alterniflora*, *Spartina anglica*, *Spartina densiflora*, *Spartina patens* (Aiton) Muhl., *Spartina ×townsendii* H. Groves & J. Groves).

This taxonomic study was prompted by difficulties encountered in determining recent herbarium collections of invasive *Spartina* from British Columbia. Existing regional taxonomic resources do not include all taxa known in the province (Hitchcock et al. 1969, Douglas et al. 2001, Kozloff 2005) since several species are new or recent invaders, and continent-wide treatments (Barkworth 2003) were found initially to be unsatisfactory to use in this taxonomically difficult grass genus. Since *Spartina* invasions are causing serious ecological damage on the Pacific coast, there has been considerable effort to develop information sources for field-based identification, probably in response to a lack of easy-to-use and easy-to-access locally relevant information in the scientific literature, in combination with an urgent need for information to facilitate *Spartina* identification by as broad a user group as possible to minimize the impacts of the invasions. These mostly emphasize vegetative characteristics easy to observe in the field, such as plant height, blade width and height, leaf color, and plant habit (e.g., Anonymous no date, 2009, Pfauth and Sytsma 1998, O’Connell 2005, Eissinger 2008) but, unfortunately, none of these are among the best taxonomic characters to identify *Spartina* species with confidence, particularly those that are closely related. Several of these resources do not clearly list authors, publication dates or places other than websites, and they do not identify the original sources (e.g., floras, the primary literature, herbarium specimens) from which the information was derived.

The purpose of this paper is to provide up-to-date taxonomic information for specimen-based identification of *Spartina* species in the Pacific Northwest. Although field-based identification of invasive *Spartina* taxa is possible, reliable determinations should be made or confirmed from specimens, as most of the diagnostic characters require magnification and careful, accurate measurements. Specimens should be deposited in herbaria, where they become part of the scientific record, are available for study by other scientists, and document the distributions of species in time and space. Voucher specimens for invasive plants such as *Spartina* are particularly important, as they provide the raw materials from which reliable and repeatable identifications can be made, and they contribute to long-term understanding of the distribution and spread of these new invaders. Unfortunately, herbaria often have relatively few specimens of weedy species, a situation recently documented for noxious weeds in Washington (Zika 2006). Accordingly, there are relatively few herbarium specimens for invasive *Spartina* taxa in the Pacific Northwest, despite considerable efforts to eradicate *Spartina* infestations (e.g., Hedge et al. 2003), effectively representing the collection of hundreds to thousands of plants.

Here, I present a taxonomic synopsis of the two native and five introduced taxa of *Spartina* known from British Columbia, Washington, and Oregon (*Spartina alterniflora*, *Spartina anglica*, *Spartina densiflora*, *Spartina gracilis* Trin., *Spartina patens*, *Spartina pectinata* Link, and *Spartina ×townsendii*). I provide a new taxonomic key for the region, nomenclature for all taxa, including previously unpublished details on several type specimens and new lectotypifications, references to published illustrations that clearly show diagnostic characters, specimen citations, and notes on how to distinguish the taxa. New descriptions are given for the closely related and morphologically similar taxa *Spartina alterniflora*, *Spartina anglica*, and *Spartina ×townsendii*, and the aggressively invading *Spartina densiflora*, which has recently appeared in British Columbia. *Spartina ×townsendii* is newly reported from British Columbia. The descriptions and keys are based on study of specimens collected within and outside the region, in consultation with the global primary and secondary taxonomic literature; these data should be useful for distinguishing the taxa wherever they occur globally, including in Alaska where *Spartina* has not been reported but is anticipated to become a problem in the future (Morgan and Sytsma 2010). I also include detailed reviews of the evolutionary origins and introduction histories of the invasive *Spartina* taxa, with a focus on their introductions in British Columbia, Washington, and Oregon. To facilitate species identification, images of exemplar herbarium specimens of each taxon are included in the main text, and images of fifty herbarium specimens (a subset of the material examined) are included as supplementary information.

### Higher-level classification of Spartina

*Spartina* is a member of the grass subfamily Chloridoideae Kunth ex Beilschm., one of six major lineages (subfamilies) in the grass PACMAD clade, which also includes the subfamilies Panicoideae Link, Arundinoideae Burmeist., Micrairoideae Pilg., Aristidoideae Caro and Danthonioideae H.P.Linder & N.P.Barker (reviewed in Saarela and Graham 2010). Chloridoideae includes some 1420 species in approximately 140 genera (Peterson et al. 2010). In the most recent classification of Chloridoideae, *Spartina* is included in the tribe *Zoyseae* Benth., a lineage characterized by glabrous culm nodes, ligules that are a line of hairs or a fringed membrane, bisexual spikelets that are laterally compressed (or terete, rarely dorsiventrally compressed), spikelets with a single floret, lower glumes nerveless or 1-veined, lemmas 1- or 3-veined and awnless, ovaries glabrous, a base chromosome number of x = 7–10, and a preference for saline habitats (Peterson et al. 2007, 2010). *Zoyseae* includes the *incertae sedis* genus *Urochondra* C.E. Hubb. and the subtribes *Zoysiinae* Benth. (one genus, *Zoysia* Willd.) and *Sporobolinae* Benth. *Sporobolinae* is characterized by spikelets oriented abaxially along the axis with the lemma facing the rachis, lemmas that are similar in texture to the glumes, paleas that are relatively long and approximately equal in length to the lemma, and free (reluctantly free in *Spartina*) pericarps (Peterson et al. 2007). *Sporobolinae* includes the large polyphyletic genus *Sporobolus* R.Br.(some 200 species; Simon et al. 2011) in which the genera *Calamovilfa* (A. Gray) Hack. ex Scribn. & Southw., *Crypsis* Aiton, *Pogoneura* Napper, and *Spartina* are embedded – a phylogenetic structure that has been identified in multiple molecular studies (e.g., Hilu and Alice 2001, Columbus et al. 2007, Peterson et al. 2010). Peterson et al. (2010) recommended future expansion of *Sporobolus* to include all of these genera, including the monophyletic *Spartina*, reflecting their origins from within the *Sporobolus* line. If (or more likely, when) these nomenclatural changes are proposed, species currently treated in *Spartina* would be treated as species of *Sporobolus*, more accurately reflecting our understanding of the evolutionary history of these grasses and their closest relatives.

### Spartina in North America

Taxonomic revisions of *Spartina* have been produced by Merrill (1902), Saint-Yves (1932) and Mobberley (1956), the latter being the most recent and comprehensive, though it does not cite the specimens examined during the study. Mobberley (1956) recognized some fifteen taxa in the genus, including three native to the Old World (*Spartina anglica*, *Spartina maritima* (Curtis) Fernald, *Spartina townsendii*), and the remainder native to the New World. There are thirteen species of *Spartina* in North America, of which nine are native (including one natural hybrid), and four are introduced (Mobberley 1956, Barkworth 2003). They are distributed in low to high intertidal marsh (brackish), inland brackish and freshwater habitats and dry prairie, and have been classified ecologically by habitat as low marsh, high marsh, and freshwater (Maricle et al. 2009). Marsh taxa include the native species *Spartina alterniflora*, *Spartina patens*, *Spartina bakeri* Merr. (sand cordgrass), *Spartina spartinae* (Trin.) Merr. (gulf cordgrass) and *Spartina foliosa* Trin. (California cordgrass), and the introduced species *Spartina anglica*, *Spartina ×townsendii* and *Spartina densiflora*. Freshwater/inland taxa include *Spartina cynosuroides* (L.) Roth (big cordgrass), *Spartina gracilis*, *Spartina pectinata*, and the hybrid taxon *Spartina ×caespitosa* A.A. Eaton (mixed cordgrass; *Spartina patens × S. pectinata*).

*Spartina* has a base chromosome number of *x* = 10, and all species are polyploids (e.g., Marchant 1963, 1986b, Ayres et al. 2008, Fortune et al. 2008, Renny-Byfield et al. 2010). Phylogenetic analyses based on two nuclear (ITS and *Waxy*) and one plastid locus identified two major lineages in the genus, one comprising the tetraploid (2*n* = 40) species *Spartina patens*, *Spartina gracilis*, *Spartina ciliata* Brongn., *Spartina pectinata*, *Spartina cynosuroides*, *Spartina bakeri*, and *Spartina arundinacea* (Thouars) Carmich., and the other comprising the hexaploid (2*n* = 60, 62) species *Spartina alterniflora*, *Spartina foliosa*, and *Spartina maritim*a (Curtis) Fernald (Baumel et al. 2002b). In this analysis one tetraploid species, *Spartina argentinensis* Parodi, was sister to the hexaploid clade, and placement of the heptaploid *Spartina densiflora* (2*n* = 70, Ayres et al. 2008, Fortune et al. 2008) differed among data partitions (Baumel et al. 2002b). A subsequent phylogenetic study, based on extensive cloning of the low copy nuclear gene *Waxy*, focused on the hexaploid taxa and revealed a more complicated phylogeny, and an allopolyploid origin for the hexaploid clade was proposed (Fortune et al. 2007). Further phylogenetic study of *Spartina densiflora* revealed the taxon to be of hybrid origin involving lineages related to the tetraploid species *Spartina arundinacea* and the hexaploid clade (Fortune et al. 2008).

## Taxonomy

### Spartina

Schreb., Gen. Pl. ed. 8[a]. 43. 1789. Type: Spartina cynosuroides (L.) Roth

http://species-id.net/wiki/Spartina

Ponceletia Thouars., Esquisse Fl. Tristan D’Acugna 36. 1808. Type: *Ponceletia arundinacea* Thouars.
Solenachne Steud. Syn. Pl. Glumac. 1: 12. 1855. Type: *Solenachne phalaroides* Steud.

Plants perennial, culms cespitose from knotty bases or solitary from conspicuous creeping rhizomes. Leaves cauline; sheaths open; ligules a line of hairs; blades flat to involute. Inflorescences with multiple branches (i.e., spikes) inserted along a main axis, branches usually alternate, appressed to spreading. Spikelets laterally compressed, one-flowered, arranged in two rows along two sides of a more or less triquetrous axis, disarticulating below the glumes. Glumes unequal, strongly keeled; lower glumes 1-veined, shorter than upper glumes and floret; upper glumes 1–6-veined, usually longer than the floret. Lemmas 1–3-veined, keeled, shorter than the paleas. Paleas 2-veined, thin and papery, longer than the lemma. Anthers 3. Styles 2. Caryopses linear. Base chromosome number, *x* = 10. Named from the Greek *spartine*, a cord made from *Spartium junceum* L.(Spanish Broom; Fabaceae), and probably applied to *Spartina* in reference to its tough leaves (Hitchcock 1951).

#### Key to native and introduced species of Spartina in British Columbia, Washington and Oregon

**Table:** 

1	Leaf blades distinctly scabrous on their margins; spikelets tightly appressed and strongly overlapping	2
–	Leaf blades glabrous on their margins (occasionally with a few scattered teeth, but teeth never abundant); spikelets weakly appressed and weakly overlapping	5
2	Culms growing in tufts (i.e., cespitose) from hard knotty bases, rhizomes absent, rarely short; branches appressed, usually not readily discernible within an inflorescence, not distinctly one-sided	*Spartina densiflora*
–	Culms growing from rhizomes; branches appressed, ascending, or spreading, readily discernible within an inflorescence, distinctly one-sided	3
3	Upper glumes distinctly awned, awns 3–8 mm long; blades 5–15 mm wide; ligules 1–3 mm long; branches pedunculate, rarely sessile	*Spartina pectinata*
–	Upper glumes unawned or short-awned, when present awns to 2 mm long; blades 0.5–8 mm wide; ligules 0.5–1 mm long; branches sessile, rarely pedunculate	4
4	Glume keels ciliate, hairs stiff, 0.5–0.8(–1) mm long; glumes with two inconspicuous lateral veins on one side of the keel; branches appressed to the main axis; most branches 3–6 mm wide; inflorescences 8–25 cm long; spikelets ovate to lanceolate; florets more or less equaling the upper glumes in length	*Spartina gracilis*
–	Glume keels scabrous, teeth 0.1–0.2 mm long; glumes with two conspicuous lateral veins on one side of the keel; branches appressed, ascending, or spreading from main axis; most branches 2–2.5 mm wide; inflorescences 3–15 cm long; spikelets linear lanceolate to ovate lanceolate; florets shorter than the upper glumes	*Spartina patens*
5	Spikelets 8–14(–16.5) mm long; branch rachises 0.4–1 mm wide between spikelets; glumes glabrous or weakly pubescent; leaf blades more or less erect, forming an angle 15–18° with the culm	*Spartina alterniflora*
–	Spikelets 14–25 mm long; branch rachises 1–2.2 mm wide between spikelets; glumes moderately to densely pubescent; leaf blades ascending to spreading, forming an angle 30–60° with the culm	6
6	Spikelets (15–)16.5–25 mm long; anthers 7–10 mm long, usually fully exserted at maturity; pollen fertile; ligules 1–3 mm long; upper glumes 3–6-veined, 13–22 mm long; glumes (weakly) moderately to densely pubescent with hairs 0.1–0.3 mm long, hairs to 0.6 mm long and usually denser proximally; calluses (1.5–)2–4.5 mm long; branches (3–)4–5(–6) mm wide	*Spartina anglica*
–	Spikelets 14–17.5 mm long; anthers 5–7(–8.5) mm long, not or incompletely exserted at maturity, indehiscent; pollen sterile; ligules 1–1.5 mm long; upper glumes 3-veined, 12.5–16.5 mm long; glumes weakly to moderately pubescent with hairs 0.1–0.2 mm long, occasionally to 0.6 mm long proximally; calluses 0.6–1.5(–2) mm long; branches (2.5–)3–4 mm wide	*Spartina ×townsendii*

### Spartina alterniflora

Loisel., Fl. Gall. ii. 719. 1807.

http://species-id.net/wiki/Spartina_alterniflora

**Appendix figure 1.**
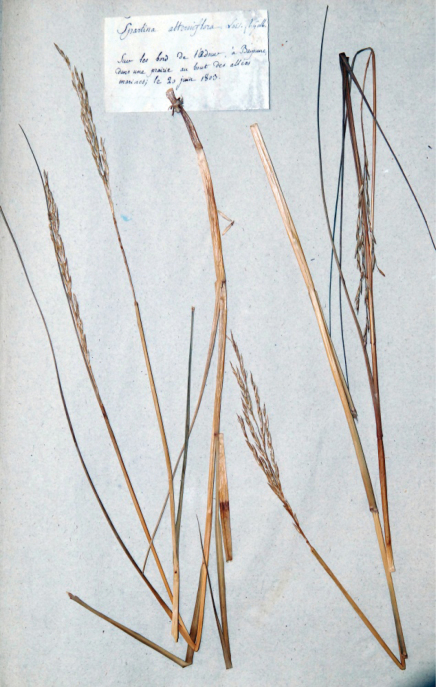
Holotype specimen of *Spartina alterniflora* (without collector, AV). Photo: Museum Requien. Specimen image reproduced with the permission of Museum Requien.

**Appendix figure 2.**
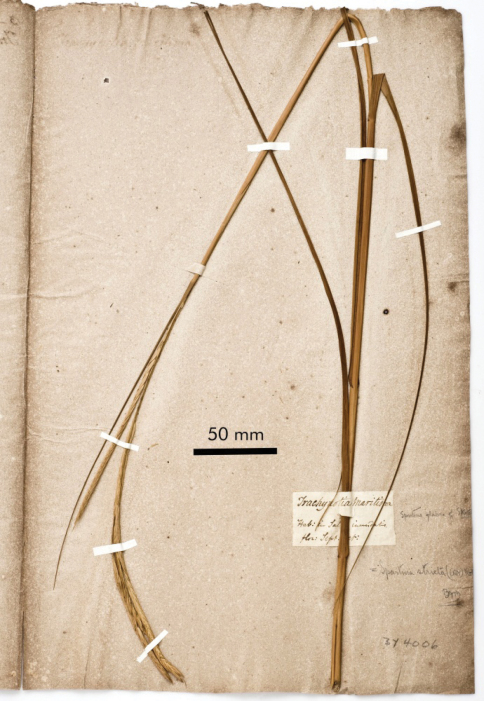
Holotype specimen of *Spartina glabra* (Elliott s.n., ChMBY4006). Photo: Sean Money, Charleston Museum.

Spartina glabra var. *alterniflora* (Loisel.) Merr., U.S.D.A. Bur. Pl. Industr. Bull. 9: 9. 1902. *Spartina maritima* var. *alterniflora* (Loisel.) St.-Yves. Candollea 5: 25, 53, pl. 2, f. 1-4, 1932. *Spartina stricta* var. *alterniflora* (Loisel.) A. Gray, Manual (ed. 2) 552. 1856. *Trachynotia alterniflora* (Loisel.) DC., Fl. Franc. (ed. 3) 5: 279. 1815. **Type**. France. Sur les bord de l’Odo[…?], à Bayonne, dans une prairie au bout des allées marines, 20 Jun 1803, n.c. (holotype: AV!, Appendix Fig. 1). Note: The location of the holotype in the herbarium (AV) at Museum Requien in Avignon, France, has not been reported previously in the literature.
Dactylis fasciculata Lam., Tabl. Encycl. 1: 180. 1791. **Type**. UNITED STATES OFAmerica. D.Richard s.n. (holotype: P [P00564318!] (hb. Lamarck); isotype: US [USA865637!] (fragm. ex P)).
Spartina glabra Muhl. ex Elliott, Sketch Bot. S. Carolina 1(1): 95, pl. 4, f. 2. 1816. *Limnetis glabra *(Muhl. ex Elliott) Eaton & Wright, Man. Bot. (ed. 8) 301. 1840. *Spartina alterniflora *var. *glabra* (Muhl. ex Elliott) Fernald, Rhodora 18: 178. 1916. *Spartina maritima* subsp. *glabra* (Muhl. ex Elliott) St.-Yves, Candollea 5: 48. 1932. *Spartina maritima* var. *glabra* (Muhl. ex Elliott) St.-Yves, Candollea 5: 24, 49, t. 1, f. b-2. 1932. *Spartina stricta* var. *glabra* (Muhl. ex Elliott) A. Gray, Manual ed. 2. 552. 1856. **Type**. United States of america.South Carolina or Georgia: S.Elliott s.n. (holotype: ChM [ChMBY4006!] (hb. Elliott), Appendix Fig. 2). Note: There is a specimen collected by S. Elliott, likely in 1810 or 1811, in the Elliott herbarium at the Charleston Museum (Charleston, South Carolina). Chauncey Beadle and Charles Boynton conducted a systematic inventory of the Elliott herbarium in the first decade of the 20th century; they determined this specimen to be Elliott’s “*Spartina glabra* of Sketch” and annotated it as such on the sheet (A.E. Sanders, personal communication 2011). This specimen is the holotype of *Spartina glabra*. Material seen by Muhlenberg is housed at PA (see Hitchcock 1932: 30).
Spartina brasiliensis Raddi, Agrostogr. Bras. 21-22. 1823. *Spartina maritima* var. *brasiliensis* (Raddi) St.-Yves, Candollea 5: 56, t. 2, f. 56. 1932. **Type.** Brazil. Rio de Janeiro, G.Raddi s.n. (holotype: PI; isotypes: FI (2 sheets), PI, US [US3376186!] (fragm. ex PI)).
Spartina glabra var. *pilosa* Merr., U.S.D.A. Bur. Pl. Industr. Bull. 9: 9. 1902. *Spartina alterniflora* var. *pilosa* (Merr.) Fernald, Rhodora 18: 179. 1916. *Spartina maritima* subvar. *pilosa* (Merr.) St.-Yves, Candollea 5: 51, t. 1. f. c-3. 1932. **Type.** UNITED STATES OF AMERICA. New Jersey: Aug 1895, F.L.Scribner s.n. (holotype: US [US81736!]). Note: Peterson (2001) identified the US specimen as an isotype, but I interpret it to be the holotype, as Merrill (1902) indicated his work was based entirely on specimens in US, and wrote type after citing this specimen in the protologue.
Spartina ×merrilli A.Chev. Bull. Soc. Bot. France 80: 787. 1933. **Type.** UNITED STATES OF AMERICA. New York: Long Island, Rockaway Point, 22 Oct 1908, E.P.Bicknell 11300 (holotype: NY [NY431515!]; isotype: PH [PH736377!] Note: Chevalier (1933: 788) lists nine specimens in the protologue, and indicates in a figure that Bicknell 11300 is the type; the NY specimen of this collection has ‘type’ handwritten on it.
Spartina maritima  subvar. *fallax* St.-Yves, Candollea 5: 57. 1932. **Type.** BRAZIL. Bahia: Salzmann s.n. [sheet A] (lectotype: P [P02249890!], here designated). Note: St-Yves (1932) listed twelve specimens (syntypes) in the protologue, including the one designated here as lectotype.

#### Description.

Culms to 250 cm tall, rhizomatous. Sheaths glabrous; ligules 1–2 mm long; blades 5–63 cm long × 3–10 mm wide at base, usually flat proximally, involute distally, divergent from stems 15–18°, adaxial and abaxial surfaces glabrous, margins smooth, rarely with occasional scabrous teeth. Inflorescences (6–)11–33 cm long × (5–)6–10(–15) mm wide at midpoint, erect, with 3–9(–12) branches; branches (4)5–15 cm long × 2–4 mm wide, appressed to main axis or ascending, rachises 0.4–1 mm wide between spikelets, extending 1–20 mm beyond terminal spikelet. Spikelets 8–14(–16.5) mm long × 1–2 mm wide, alternate, weakly appressed, weakly or moderately overlapping, calluses 0.5–1.5 mm long. Glumes glabrous or weakly pubescent, when present hairs to 0.2 mm long, proximal hairs sometimes denser and longer to 0.5 mm, keels glabrous or ciliate, when present hairs to 0.3 mm long, margins glabrous; lower glumes 4–9 mm long × 0.2–0.5 mm wide, 1-veined, tips acute; upper glumes 7–14 mm long × 1–1.2 mm wide, 5–7-veined, tips acuminate or obtuse. Lemmas 7–12 mm long, glabrous or scabrous; paleas exceeding lemmas by up to 1 mm; anthers 3–6 mm long, yellow, exserted at maturity, dehiscent, pollen fertile. 2*n* = 62 (Marchant 1963, 1968b).

#### Common name.

Smooth cordgrass, Atlantic cordgrass, Atlantic smooth cordgrass.

#### Etymology.

The epithet *alterniflora* means alternating flowers.

#### Illustrations.

Hitchcock 1935:492, Hitchcock 1951:512, Hitchcock et al. 1969:706, Holmgren et al. 1998:749, Barkworth 2003:245, Cope and Gray 2009:543, Scholz et al. 2009:172.

#### Distribution.

Native to the Atlantic and Gulf coasts of North America from Newfoundland and Labrador, Canada, to Texas, U.S.A. (Mobberley 1956, Barkworth 2003), and Tamaulipas and Veracruz, Mexico (Mora-Olivo and Valdés-Reyna 2011). Introduced in England, France, and Spain (Campos et al. 2004), the west coast of the United States (Washington, Oregon, California), New Zealand (Partridge 1987), and China (Bixing and Phillips 2006, An et al. 2007, Scholz et al. 2009).

#### Comments.

*Spartina alterniflora* is often misspelled in the literature as “Spartina alternifolia”. *Spartina alterniflora* was described from Bayonne in southwestern France (Loiseleur-Deslongchamps 1807), where it has been introduced for over two centuries. The taxon was collected in France as early as 1803, and was known from southern England (Southampton Water) by 1829; it is presumed to have been introduced from North America to these European coastal regions in shipping ballast (see Bromfield 1836, Marchant 1968). *Spartina alterniflora* is one of the parents of the sterile F1 hybrid *Spartina ×townsendii* (see notes under this taxon), which was formed when this introduced species hybridized with the European Atlantic coast native species *Spartina maritima*. By 1969 *Spartina alterniflora* was extremely rare in Southampton Water, known from only a single locality (Marchant and Goodman 1969a, 1969b), and it remains rare in Britain (Cope and Gray 2009).

Several introductions of *Spartina alterniflora* have occurred along the west coast of North America where it is not native. The species was reported in 1945 from a single estuarial location in Willapa Bay, Washington, where occasional plants had been observed since around 1911, and thought to have been introduced in the early twentieth century with oyster culture (Scheffer 1945). The earliest known record documenting *Spartina alterniflora* at Willapa Bay is a photograph taken in the early 1940s by T. Scheffer, housed at the California Academy of Sciences Herbarium (Civille et al. 2005). Based on a review of historical records of the oyster trade, Civille et al. (2005) concluded that *Spartina alterniflora* was probably introduced to Willapa Bay between 1893 and the late 1910s via seeds attached to oysters during their railway transport from the Atlantic coast to the Pacific coast. Since the mid-twentieth century *Spartina alterniflora* has expanded dramatically in Willapa Bay and northwards to Grays Harbour and the Copalis River estuary in Washington (Stiller and Denton 1995), radically transforming these tidal ecosystems from mudflats to dense cordgrass swards (e.g., Feist and Simenstad 2000, Davis et al. 2004a, 2004b). Civille et al. (2005) used historical records and remote sensing to reconstruct the dramatic expansion of *Spartina alterniflora* that occurred in Willapa Bay between 1945 and 2000, and concluded that its rapid colonization likely originated from multiple introductions over several decades, as opposed to the long-standing hypothesis of post-establishment colonization following a single introduction to the region (e.g., Scheffer 1945, Stiller and Denton 1995, Feist and Simenstad 2000). Considerable research has been conducted on understanding the invasion dynamics of *Spartina alterniflora* in Willapa Bay (e.g., Davis et al. 2004a, 2004b, Taylor and Hastings 2004, Grosholz et al. 2009), and substantial efforts and resources have been directed at its management, control and eradication (e.g., Grevstad et al. 2003, Taylor and Hastings 2004). *Spartina alterniflora* is also known from the southern end of Padilla Bay in northwestern Washington, where it was apparently introduced independently between 1941 and 1945 to aid in beach stabilization (see Wiggins and Binney 1987, Riggs 1992). Stiller and Denton (1995) confirmed the genetic distinctiveness of this stand compared with the Willapa Bay population, supporting its putative independent origin.

In Oregon, *Spartina alterniflora* has been reported from the Siuslaw River estuary and Coos Bay (Howard 2005; Wilson et al. 2012). In California *Spartina alterniflora* occurs in southeastern San Francisco Bay (Alameda Creek Flood Control Channel and along the shoreline some three km south) where it was planted for restoration in the late 1970s (Spicher and Josselyn 1985, Ayres et al. 2003). By some 20 years later it had spread considerably, and was known from seven different areas in San Francisco Bay (Callaway and Josselyn 1992). *Spartina alterniflora* has also been reported from Bolina’s Lagoon and Point Reye’s National Seashore north of San Francisco Bay; these occurrences are thought to have originated from floating seed originating in San Francisco Bay, but this hypothesis has not been confirmed (Ayres et al. 2003).

The only native *Spartina* taxon in California is *Spartina foliosa*, and by the 1990s it was known that *Spartina alterniflora* was in the process of competitively excluding *Spartina foliosa* (Callaway and Josselyn 1992). Substantial evidence has accumulated indicating that the two taxa have successfully interbred producing a highly invasive fertile hybrid capable of backcrossing with its parental taxa, resulting in a genetically heterogeneous hybrid cordgrass swarm (i.e., *Spartina alterniflor*a × *Spartina foliosa*) that is much more invasive than either parental taxon (Daehler and Strong 1997, Ayres et al. 1999, 2003, 2004, Anttila et al. 2000). Morphological variation of these hybrids is not well characterized in the literature.

*Spartina alterniflora* is not known from British Columbia, Canada. Daehler and Strong (1996) identified estuaries along the Pacific coast extending to the Canada / United States border, which they predicted to be potentially vulnerable to invasion by *Spartina* species, including *Spartina alterniflora*. Workers should be aware of the potential for its invasion and keep watch for the species in British Columbia. In a recent study of saltmarsh diatoms in central mainland British Columbia some 40 km northeast of Vancouver Island, Roe et al. (2009) reported *Spartina alterniflora* as a dominant species in low and mid marsh components of Waump (51°11'15N, 126°55'15W) and Wawwat’l saltmarshes (51°11'36N, 126°40'5W) in Seymour Inlet, based on fieldwork conducted in 2002. If the species is correctly identified as *Spartina alterniflora* in this study, this would be the first known site of the taxon in British Columbia. Alternatively, the taxon may be *Spartina anglica*, but it is not possible to further confirm its identification based on the study (voucher specimens are not mentioned). *Spartina* has not otherwise been reported from Seymour Inlet. This region should be re-visited for further field study and collection of voucher specimens. Whatever the identity of the species is, this report suggests that by 2002 invasive *Spartina* was present in coastal British Columbia at sites further north than those first documented in 2003 near the international border south of Vancouver.

#### Morphology.

**Figure 1.**
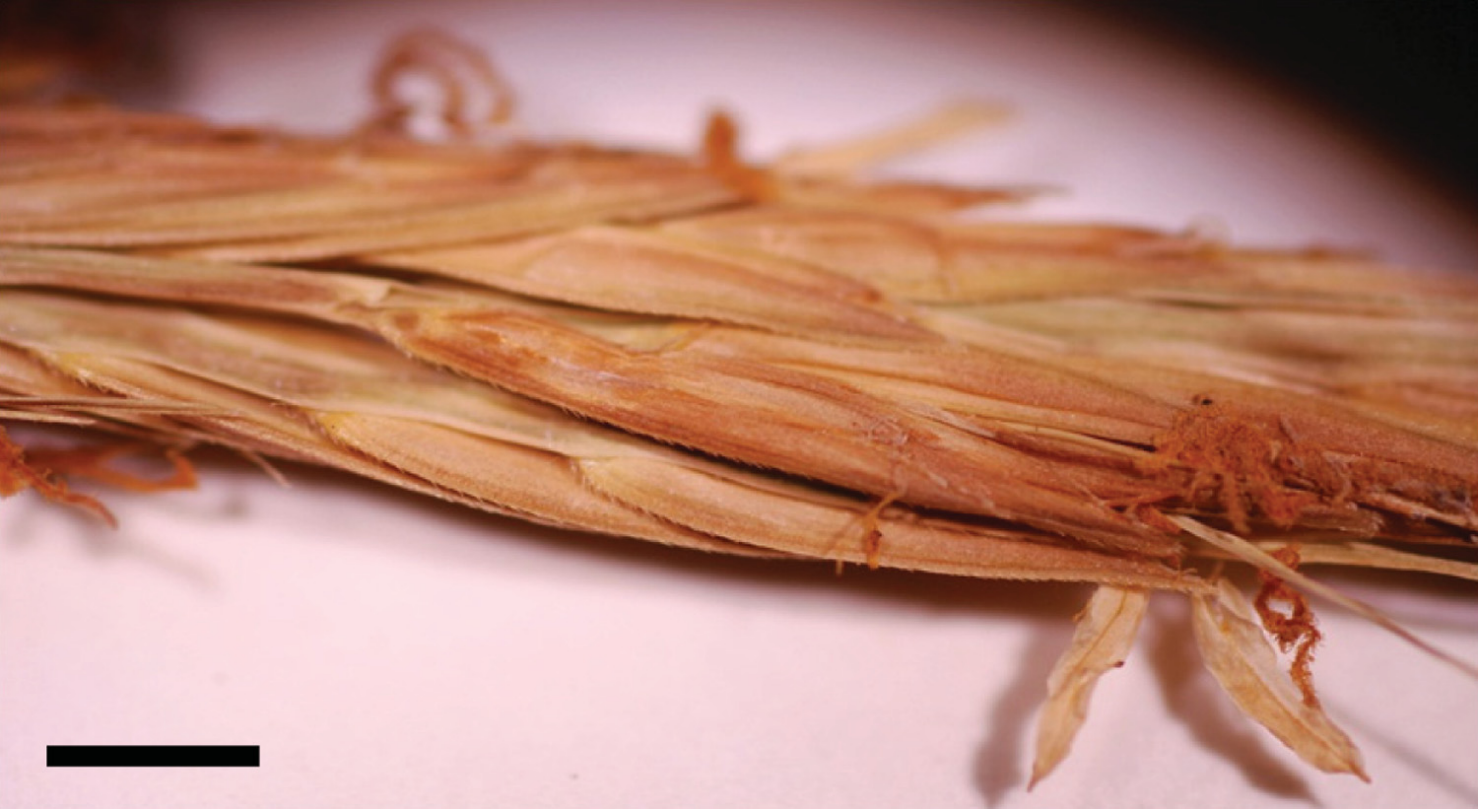
Spikelets of *Spartina alterniflora* (U.S.A.: Washington, Pacific Co., Zika 18935, WTU). Bar = 3 mm. Photo: J.M. Saarela.

**Figure 2.**
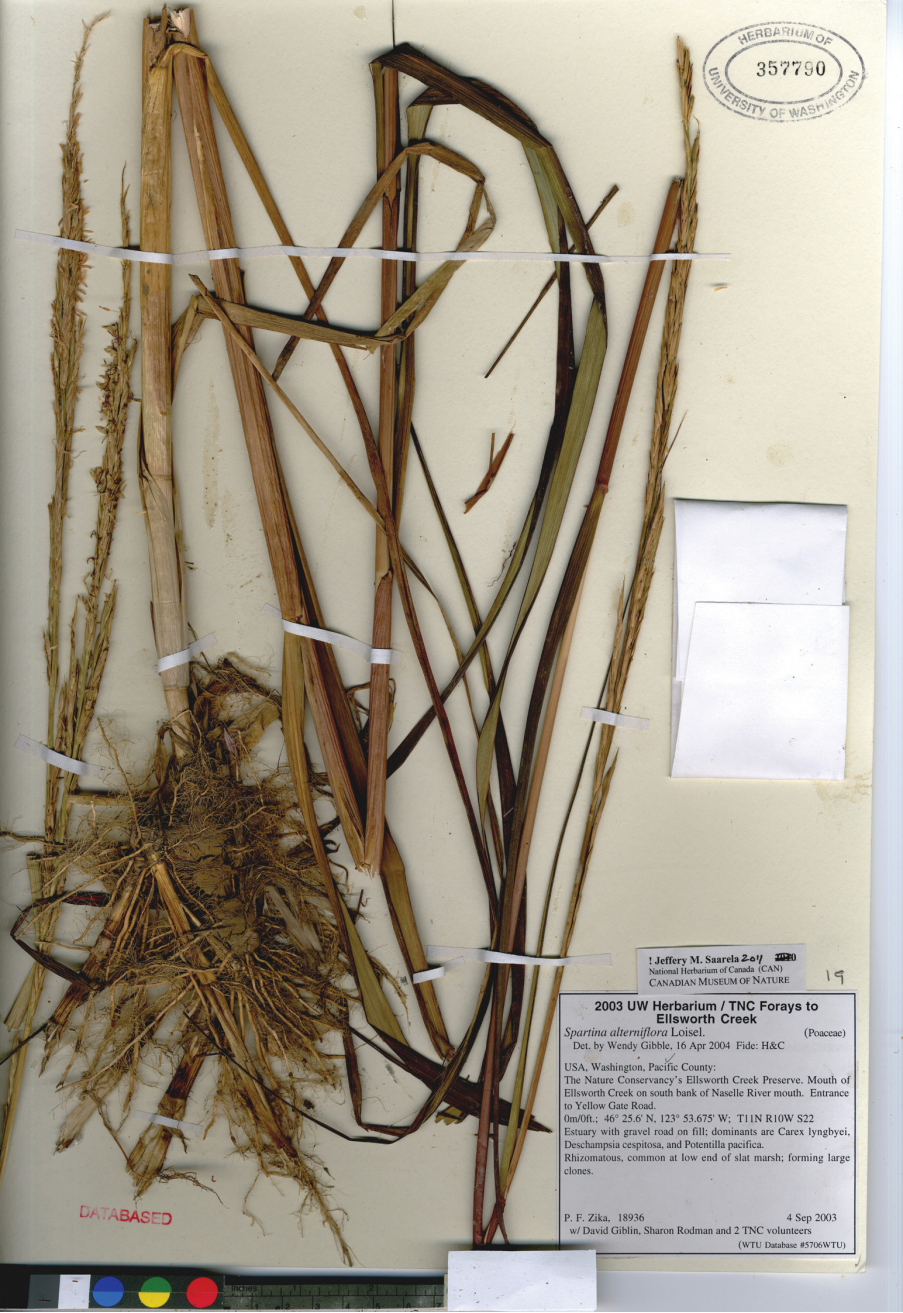
Photograph of a specimen of *Spartina alterniflora* collected at Ellsworth Creek Preserve, Pacific County, Washington, where the species is introduced (Zika et al. 18936, WTU). Image published with the permission of University of Washington Herbarium, Burke Museum.

*Spartina alterniflora* and the European species *Spartina maritima* are the parents of the sterile F1 hybrid *Spartina ×townsendii*; unsurprisingly, *Spartina alterniflora* is morphologically similar to *Spartina ×townsendii* and the amphidiploid *Spartina anglica*. It can be distinguished from these taxa by its shorter spikelets [8–14(–16.5) mm vs. 14–25 mm], narrower branch rachises [0.4–1 mm wide between spikelets vs. 1–2.2 mm wide], glumes glabrous or weakly pubescent [vs. glumes weakly to densely pubescent], and leaf blades erect, forming an angle of 15–18° with the culm [vs. leaf blades spreading, forming an angle of 30–60° with the culm]. Spikelets of *Spartina alterniflora* are shown in Fig. 1, and an exemplar specimen is shown in Fig. 2. Glumes in *Spartina alterniflora* vary from glabrous to pubescent (details on this variation are given in Mobberley 1956). In individuals with glabrous glumes, this is a good character for distinguishing *Spartina alterniflora* from *Spartina ×townsendii* and *Spartina anglica*, which consistently have pubescent glumes, but the character is more difficult to interpret and less reliable in *Spartina alterniflora* individuals with pubescent glumes. *Spartina alterniflora* also tends to have narrower inflorescences than *Spartina ×townsendii* and *Spartina anglica* [(5–)6–10(–15) mm wide vs. 7–25 mm wide], but there is considerable overlap in this character. Recent taxonomic keys (Barkworth 2003, Kozloff 2005) distinguishing these three taxa emphasize variation in number of panicle branches (3–25 in *Spartina alterniflora* vs. 1–12 in the other taxa). I found this character unreliable in material examined here, and do not include it in the key. *Spartina alterniflora* can further be distinguished from *Spartina anglica* by its shorter anthers [3–6 mm long vs. 7–10 mm long], and from *Spartina ×townsendii* by its fully exserted, dehiscent anthers at anthesis and fertile pollen [vs. anthers not or incompletely exserted, indehiscent and sterile pollen]. *Spartina alterniflora* is readily distinguished from *Spartina densiflora*, *Spartina gracilis*, *Spartina patens* and *Spartina pectinata* by it glabrous leaf blade margins [vs. scabrous leaf blade margins].

There is considerable morphological variation in *Spartina alterniflora* throughout its native range with northern plants from Canada and Maine tending to have looser inflorescences, weakly overlapping spikelets, and less glume pubescence, and southern plants tighter inflorescences, more strongly overlapping spikelets, and more pubescent glumes. This variation has been recognized taxonomically in the past at the species and infraspecific levels; however, Mobberley (1956) demonstrated the variation to be clinal and therefore taxonomically inconsistent, and only a single taxon is now generally accepted (e.g., Barkworth 2003). Specimens examined from the Pacific coast in Washington have moderately overlapping spikelets.

#### Specimens examined.

**Appendix figure 3.**
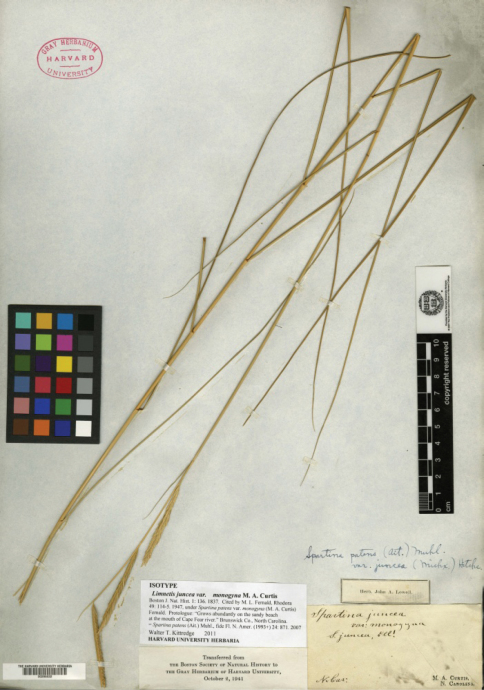
Holotype specimen of *Limnetis juncea* var. *monogyna* (Curtis s.n., GH [GH00295532]). Photo: Gray Herbarium of Harvard University Herbarium.

**CANADA. New Brunswick:** Charlotte Co.:E of Biological Station, St. Andrews, 45°04'N, 67°03'W, Aug 1929, M.O.Malte 798/29 (CAN [CAN33914]); Grand Manan, Ross Island, 44°42'N, 66°48'W, 10 Aug 1927, C.A.Weatherby & U.F.Weatherby 5784 (CAN [CAN33957]); Grand Manan, Thoroughfare, 44°42'N, 66°48'W, 14 Aug 1944, C.A.Weatherby & U.F.Weatherby 7314 (CAN [CAN33913]). Gloucester Co.: Bathurst and vicinity, 47°37'N, 65°39'W, 2 Aug 1926, M.O.Malte 732 (CAN [CAN124678]). Restigouche Co.: Dalhousie, 48°04'N, 66°22'W, 4 Aug 1955, H.J.Scoggan 12682 (CAN [CAN240149]). Saint John Co.: St. John, 45°16'N, 66°04'W, 17 Aug 1877, J.Macoun 28971 (CAN [CAN33915]); Saints’ Rest Beach, on W side of St. John, St. John Harbour, NE of Lorneville, ca. 45°15'N, 66°02'W, 24 Aug 1975, P.M.Catling & S.M.McKay s.n. (CAN [CAN396499]). Westmoreland Co.: Moncton, 46°08'N, 64°46'W, 18 Sep 1912, M.O.Malte 108312 (CAN [CAN206832]); 1 mi E of Cape Bimet, 5 mi E of Shediac, 46°14'N, 64°27'W, 7 Aug 1981, M.Shchepanek & A.Dugal 3615 (CAN [CAN474764]). **Newfoundland and Labrador:** Chapel Island, Rocky Point, 3 mi SE of Summerford, 49°28'N, 54°45'W, 13 Aug 1977, M.J.Shchepanek & D.White 3051 (CAN [CAN436102]). St. Georges District**,** Stephenville Crossing, 48°31'N; 58°25'W, 4 Aug 1986, L.Brouillet & I.Saucier 86178 (CAN [CAN546706]); St. Georges, 48°31'11"N, 58°54'51"W, M.L.Fernald, K.M.Wiegand & J.Kittredge 2598 (CAN [CAN33900]); St. Georges, 48°31'11"N, 58°54'51"W, 4 Aug 1986, L.Brouillet & I.Saucier 86173 (CAN [CAN546843]). Port-au-Port District,West Bay Center, 4 Aug 1986, I.Saucier & A.Leduc 86185 (CAN [CAN546845]). **Nova Scotia:** Annapolis Co.:Granville, 44°47'02"N, 65°26'59"W, 18 Jul 1921, M.L.Fernald & N.C.Fassett 23294 (CAN [CAN33905]). Cape Breton Co.: Louisburg, Cape Breton, 18 Aug 1898, J.Macoun 21129 (CAN [CAN33902]). Digby Co.: Meteghan River, Clare Municipality, 44°13'N, 66°08'W, 27 Jul 1975, A.W.Dugal 75-66 (CAN [CAN475739]); Sissiboo River, Weymouth, 44°24'44"N, 65°59'43"W, 21 Aug 1920, M.L.Fernald, C.H.Bissell, C.B.Graves, B.Long & D.H.Linder 19972 (CAN [CAN33903]); Digby, 44°37'20"N, 65°45'38"W, 27 Aug 1910, J.Macoun 82104 (CAN [CAN33901]). Guysborough Co.:Canso, 45°20'12"N, 60°59'40"W, 15 Aug 1901, J.Fowler s.n. (CAN [CAN390991]); Canso, 45°20'12"N, 60°59'40"W, 1416 Aug 1930, J.Rousseau 35509 (CAN [CAN33907]); Guysborough, 45°23'N, 61°29'57"W, 6–7 Aug 1930, J.Rousseau 35357 (CAN [CAN33908]). Kings Co.: Avonport, 45°06'01"N, 64°15'27"W, 23 Jul 1957, H.J.Scoggan 13849 (CAN [CAN255571]). Lunenburg Co.: LaHave River, 44°17'37"N, 64°21'27"W, 6 Aug 1910, J.Macoun 82103 (CAN [CAN33904]). Pictou Co.: Pictou, 45°40'33"N, 62°42'33"W, 12 Aug 1880, McKay 28969 (CAN [CAN33906]); same locality, 31 Jul 1880, McKay 28972 (CAN [CAN33910]). Richmond Co.: Cape Breton Island, Richmond Municipality, Fullers River Salt Marsh, 3 km W of Fourchu, off Hwy. 327, 45°43'N, 60°18'W, M.J.Shchepanek & A.W.Dugal 6419 (CAN [CAN521694]). Queens Co.: N, end of Summerville Beach, Summerville Center, 43°57'N, 64°49'W, 28 Sep 1979, D.F.Brunton & H.L.Dickson 2092 (CAN [CAN452659]). Yarmouth Co.: Port Maitland, 43°59'03"N, 66°09'03"W, 24 Aug 1913, M.O.Malte s.n. (CAN [CAN206823]); Wedgeport, 43°42'58"N, 65°58'45"W, 18 Jul 1953, W.L.Klawe 1204 (CAN [CAN298544]); Lower Argyle, 43°43'44"N, 65°50'01"W, 11 Aug 1920, M.L.Fernald, C.H.Bissell, C.B.Graves, B.Long, D.H.Linder 19971 (CAN [CAN33909]). **Prince Edward Island:** Brackley Point, 46°23'N, 63°11'W, 4 Aug 1888, J.Macoun 28968 (CAN); Prince Co.: Tignish, 46°57'N, 64°02'W, 6 Aug 1912, M.L.Fernald, B.Long & H.St.John 6877 (CAN). Queens Co.: ¼ mi E of Pond Point, Long Creek salt marsh, 56°03'N, 62°57'W, M.Shchepanek & A.Dugal 4128 (CAN). **Quebec:** Bas-Saint-Laurent Region, Rocher blanc, 48°25'19"N, 68°36'24"W, 19 Jul 1949, Fr.Claude s.n. (CAN [CAN388720]); Cap a la Carre, St. Fabien, Gaspé, 48°17'N, 68°52'W, 9 Aug 1970, J.K.Morton NA3907 (CAN [CAN359898]); Cacouna Harbour development ca. 8 km NE of Rivière-du-Loup, 47°55'N, 69°30'W, 12 Aug 1980, D.F.Brunton 2519 (CAN [CAN455714]); Notre-Dame-du-Portage, 47°46'N, 69°37'W, 30 Aug 1970, G.Lemieux 13633 (CAN [CAN444192]); Rankin Point near Kamouraska, 24 Aug 1947, J.H.Soper & D.A.Fraser 3650 (CAN [CAN257673]). Capitale-Nationale Region, Baie-St.-Paul, 47°25'N, 71°20'W, 29 Jul 1984, S.G.Aiken 2962 (CAN [CAN484859]); Murray Bay, 47°38'60"N, 70°07'60"W, 14 Aug 1905, J.Macoun 68994 (CAN [CAN33921]). Côte-Nord Region, Anticosti Island, Ellis Bay, 49°30'N, 63°00'W, 7 Sep 1883, J.Macoun 28970 (CAN [CAN33920]); Betchwan [sic] [Betchouane], 50°14'27"N, 63°11'01"W, 25 Aug 1928, H.F.Lewis 130615 (CAN [CAN33919]); Havre des Canadiens, Natashquan, 50°10'59"N, 61°49'W, 7 Sep 1915, H.St.John 90141 (CAN [CAN33922]). Gaspésie–Îles-de-la-Madeleine Region, Parc de Forillon, Penouille, 48°51'N, 64°26'W, 17 Jul 1971, M.M.Grandtner G158 (CAN); Magdalen Islands, Coffin Island, East Cape, 47°33'N, 61°31'W, 17 Aug 1912, M.L.Fernald, B.Long & H.St.John 6878 (CAN [CAN33918]); Coin du Banc, 48°33'28"N, 64°17'34"W, 31 Jul 1939, H.J.Scoggan 646 (CAN [CAN33916]); House Harbour, Alright Island, 47°25'59"N, 61°46'W, 17 Aug 1917, F.Johansen s.n. (CAN [CAN33917]); Carleton, 48°05'N, 66°08'W, 11 Aug 1930, F.Marie-Victorin, F.Rolland-Germain & E.Jacques 33291 (CAN [CAN513103]); Gaspé Peninsula, Bonaventure River, 48°03'N, 65°29'W, 12 Aug 1940, H.J.Scoggan 1214 (CAN [CAN220299]); près du pont de la Grosse Ile, Iles-de-la-Madeleine, 47°37'N, 61°33'W, 0.3 m, 7 Aug 1966, M.M.Grandtner 10801-V (CAN [CAN519301]). **United States of America. Connecticut**: New London Co.: Norwich, 41°31'N, 72°04'W, G.R.Lumsden s.n., 11 Aug 1885 (CAN [CAN162186]). **Massachusetts:** Barnstable Co.: Provincetown, Provincetown Harbor, 42°02'N, 70°10'W, 9 Oct 1988, S.G.Aiken & S.R.Johnstone 88-488 (CAN). Essex Co.: salt marsh between Briar Neck and Bass Rocks, Gloucester, 42°36'N, 70°39'W, 26 Aug 1945, L.B.Smith 1318 (CAN [CAN162185]); same locality, 42°36'N, 70°39'W, 19 Aug 1945, L.B.Smith 1317 (CAN [CAN162182]); Salem, 42°31'N, 70°53'W, 188-, J.Sears 47174 (CAN [CAN162187]). **Maryland:** seashore, Sep 1863, Wm.M.Canby s.n. (CAN [CAN162184, CAN162183]). **Washington:** Skagit Co.: Dike Island in Padilla Bay, 7 mi W of Mt. Vernon, 19 Nov 1964, R.G.Jeffrey 64-1 (US [US2580778]). Pacific Co.: Willapa Refuge, tide water marsh, 28 Sep 1945, M.L.Hinshaw s.n. (US [US1867453]); Willapa Bay, W and N Long Island, some five small patches, scattered, apparently spreading, 10 Aug 1942, W.G.McFarland s.n. (US [US2436000]); T1N R10W, Section 22, mouth of Naselle River at SE end of Hwy. 101 bridge, 46.4291°N, 123.9078°W, 16 Sep 2000, C.L.Maxwell 1575 (WTU [WTU342828], Suppl. Fig. 1); The Nature Conservancy’s Ellsworth Creek Preserve, mouth of Ellsworth Creek on S bank of Naselle River mouth, entrance to Yellow Gate Road, T11N R10W S22, 46°25.6'N, 123°53.675'W, 0 m, 4 Sep 2003, P.F.Zika, D.Giblin, S.Rodman et al. 18936 (WTU [WTU357790], Fig. 2); Willapa Bay, sea level, 15 Sep 1994, W.Lebovitz s.n. (WTU [WTU344373], Suppl. Fig. 2); below Bruceport County Park campground, T14N R10W S22, 46°41.4'N, 123°53.1'W, 3 Sep 2003, P.F.Zika 18935 (WTU [WTU371783], Suppl. Fig. 3). **England.** Hampshire Co.: Southampton, 50°53'49"N, 01°24'15"W, s.d., Dalington s.n. (CAN [CAN134028]). Isle of Wight Co.:Isle of Wight, ca. 50°40'51"N, 01°16'51"W, 9 Oct 1871, F.Stratton s.n. (CAN). **France.** Bayonne, 43°29'N, 01°28'W, s.d., collector illegible (CAN [CAN134029]); same locality, 43°29'N, 01°28'W, Sep 1899, E.Mouillefarine s.n. (CAN [CAN560721]); Biarritz, 43°29'N, 01°33'W, Oliver s.n. (CAN [CAN421008]); Landes, near Capbreton, 43°38'N, 01°25'E, 24 Jun 1954, A.E.Porsild 18851 (CAN [CAN244668]).

### Spartina anglica

C.E. Hubb., Bot. J. Linn. Soc. 76(4): 364. 1978.

http://species-id.net/wiki/Spartina_anglica

Spartina townsendii  var. *anglica *(C.E. Hubb.) Lambinon & Maquet, Nouv. Fl. Belgique, Luxembourg, N. France (ed. 3) 923: 1983. **Type.** United Kingdom. West Sussex: Bosham, fruiging shore on mud-flats and muddy shingle, extending into water of Chichester Harbour, forming extensive masses, 17 Aug 1968, C.E.Hubbard S.17868A, sheet II (lectotype:K [K000710270!], here designated; isotypes: K [K000710269!], L [L100190!], US[US2907471!]). Note:The collection designated as the holotype by Hubbard (1978), *Hubbard S.17868A*, is mounted on two sheets at K: K000710269 and K000710270. The latter is clearly labeled ‘sheet II’, but the former is not reciprocally cross-labeled as ‘sheet I’ therefore the two sheets are duplicates according to the Code. The specimen indicated as ‘sheet II” is more robust, and is here designated as the lectotype.

#### Description.

Culms 32–104 cm tall, thick, fleshy,rhizomatous, forming clumps and dense swards. Sheaths glabrous, occasionally with short, scattered hairs, when present hairs to 0.2 mm long; ligules 1–3 mm long; blades 6–45 cm long × 4–10 mm wide, flat proximally, moderately to strongly involute distally, divergent 30–60° from culms, adaxial surfaces glabrous, occasionally sparsely pubescent proximally, when present hairs to 0.2 mm long, abaxial surfaces glabrous, occasionally sparsely pubescent proximally, when present hairs to 0.5 mm long, margins smooth. Inflorescences 12–21.5(–31.5) cm long × 7–25 mm wide at midpoint, erect, with (2)3–5(–11) branches; branches (7–)8–15(–20) cm long × (3–)4–5(–6) mm wide, appressed to main axis or ascending; rachises 1–2.2 mm wide between spikelets, extending 2–20 mm beyond the distal spikelet, glabrous, margins glabrous, occasionally sparsely pubescent, when present the hairs 0.2–0.4 mm long. Spikelets (15–)16.5–25 mm long × 1.8–2.5(–2.8) mm wide, weakly appressed, weakly overlapping, calluses (1.5–)2–4.5 mm long. Glumes (weakly) moderately or densely pubescent, hairs 0.1–0.3 mm long, hairs usually denser and to 0.6 mm long proximally; keels scabrous or ciliate, hairs to 0.5 mm long; lower glumes 8–14 mm long × 0.5–0.7 mm wide, 1-veined, tips acute or obtuse; upper glumes 13–22 mm long × 1–1.5 mm wide, 3–6 veined, tips obtuse or acute; lemmas 11–17 mm long, 1–3-veined, appressed pubescent distally, glabrous proximally, margins membranous; paleas exceeding lemmas by 1–2 mm; anthers 7–10 mm long, yellowish, usually fully exserted at maturity, dehiscent, pollen fertile. 2*n* = 120, 122, 124 (Marchant 1963, 1968b).

#### Common name.

English cordgrass; common cordgrass.

#### Etymology.

The Latin epithet *anglica* means English, given to the species in reference to England, its place of origin.

#### Illustrations.

Barkworth 2003:248, Cope and Gray 2009:551.

#### Distribution.

Britain, China (An et al. 2007, Bixing and Phillips 2006), Australia (Tasmania, Victoria, Kriwoken and Hedge 2000), New Zealand (Partridge 1987), France (western coastal Brittany, Baumel et al. 2001), United States of America (Washington, California), and Canada (British Columbia).

#### Comments.

*Spartina anglica* is an amphidiploid taxon that arose in Britain in the 18^th^ century from chromosome doubling of the sterile F1 hybrid taxon *Spartina ×townsendii* (see the discussion under that species for details, and reviews in Marchant 1968 and Gray et al. 1991). The origin of *Spartina anglica* is a remarkable and well-studied example of allopolyploid evolution.

*Spartina anglica* is a problematic invasive species in coastal areas of western North America, and has been present on the continent for over fifty years. In the United States it is known from Washington and California. It was planted in Puget Sound, Washington in 1961 (Spicher and Josselyn 1985, Frenkel 1987) to provide forage for cattle and to stabilize a dike system (Hacker et al. 2001). By 1997, it had expanded substantially in Puget Sound, occurring in 73 sites and covering 397 solid hectares of intertidal habitat (Hacker et al. 2001, see Hedge et al. 2003). Eradication efforts in this region began in 1997 (Hacker et al. 2001). In 1977 *Spartina anglica* was deliberately transplanted from Puget Sound to California where it was introduced at Creekside Park Marsh in San Francisco Bay, Marin County (Spicher and Josselyn 1985), and it persists at this single location in the state (e.g., Grijalva et al. 2006, San Francisco Estuary Invasive Spartina Project 2012). *Spartina anglica* is mentioned, but not treated in Baird and Thieret (2012).

**Figure 3.**
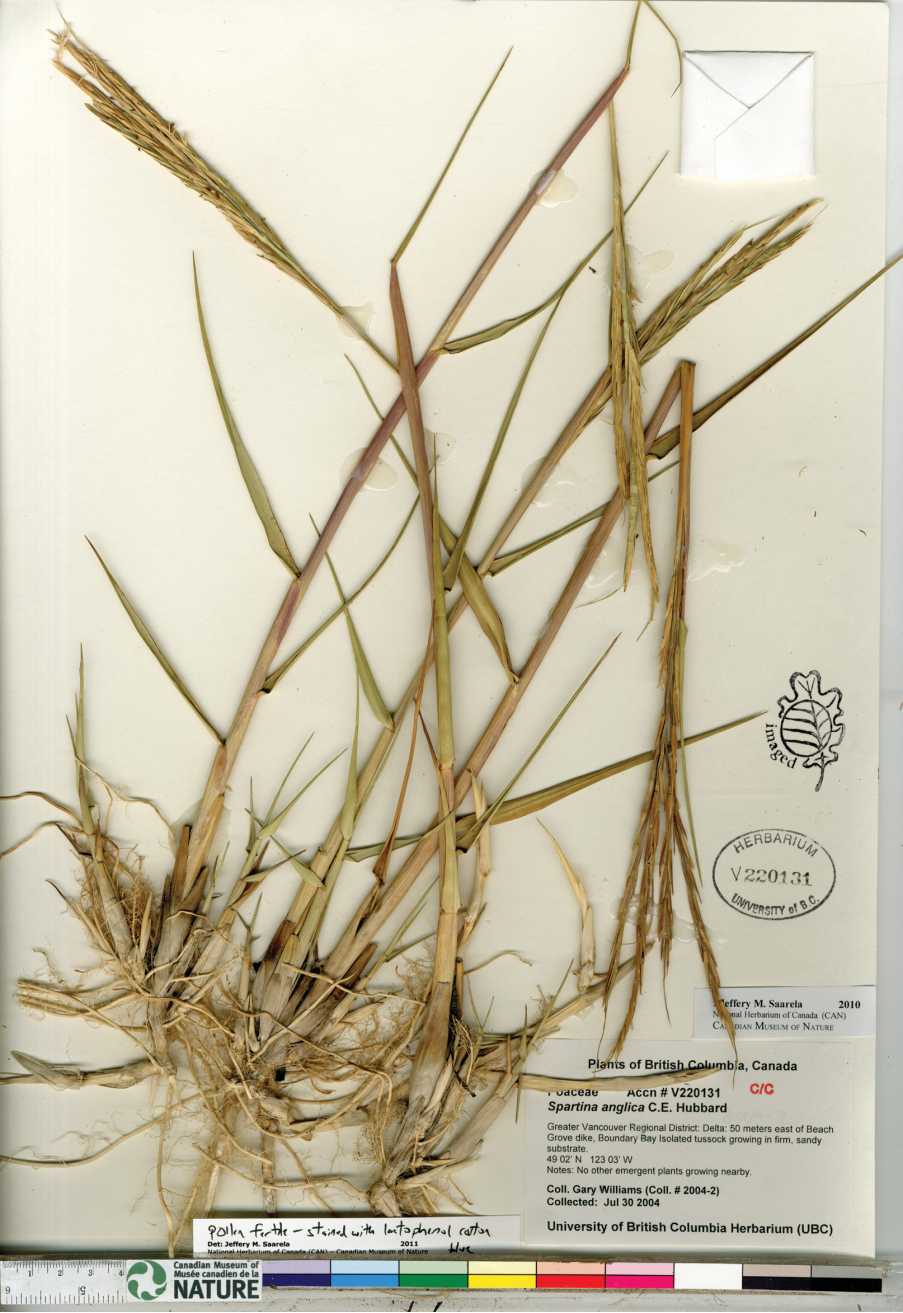
Photograph of a specimen of *Spartina anglica* collected in Boundary Bay, south of Vancouver, British Columbia (Williams 2004-2, UBC). Image published with the permission of the University of British Columbia Herbarium, Beaty Biodiversity Museum.

*Spartina anglica* was discovered in British Columbia in 2003 on Roberts Banks in the Fraser River estuary and in Boundary Bay along the British Columbia and Washington border (Williams 2004). Williams (2004) noted the infestation to be in its early stages in 2003, and estimated the species to have arrived in the area some three to five years earlier. Additional collections were made in 2004 in Boundary Bay (Lim s.n., V-two specimens) and on Roberts Banks (Williams 2004-1, 2004-2 [Fig. 3], 2004-3). Following this initial documentation of *Spartina anglica* in British Columbia, extensive field surveys and mapping exercises were undertaken to determine the extent of the species at Roberts Bank and in Boundary Bay, and an eradication plan was implemented (Dresen et al. 2010; Williams et al. 2010). Despite removal and eradication efforts, by 2009 *Spartina anglica* had increased dramatically in these regions (Ducks Unlimited Canada 2010). It is currently reproducing by seed production and vegetatively by outward clonal growth forming meadows and by drifting plant parts (R. Knight, personal communication 2010). There are only a few herbarium collections documenting *Spartina anglica* in the province, collected by G. Williams and P. Lim when the taxon was first found. Additional collections should be made at these and other sites where the species is found, to properly document its continued existence and spread.

#### Morphology.

The description here is based on collections from Washington, Oregon, and British Columbia, and Old World material housed at CAN and UBC (see Specimens Examined). *Spartina anglica* is morphologically similar to *Spartina ×townsendii*, and the two can be challenging to distinguish. Differences between these species were characterized in detail by Marchant (1968a), and my observations here are in general accordance with his findings. Mobberley (1956) commented only briefly on their differences in his treatment of *Spartina ×townsendii*, the name under which both ploidal forms of the taxon were recognized at the time. Only a few taxonomic keys in North America include both taxa (Barkworth 2003, Kozloff 2005), emphasizing variation in ligule length, anther length, and anther dehiscence for identification. In addition to these characters, the key presented here includes spikelet length, upper glume venation, and upper glume length.

**Figure 4.**
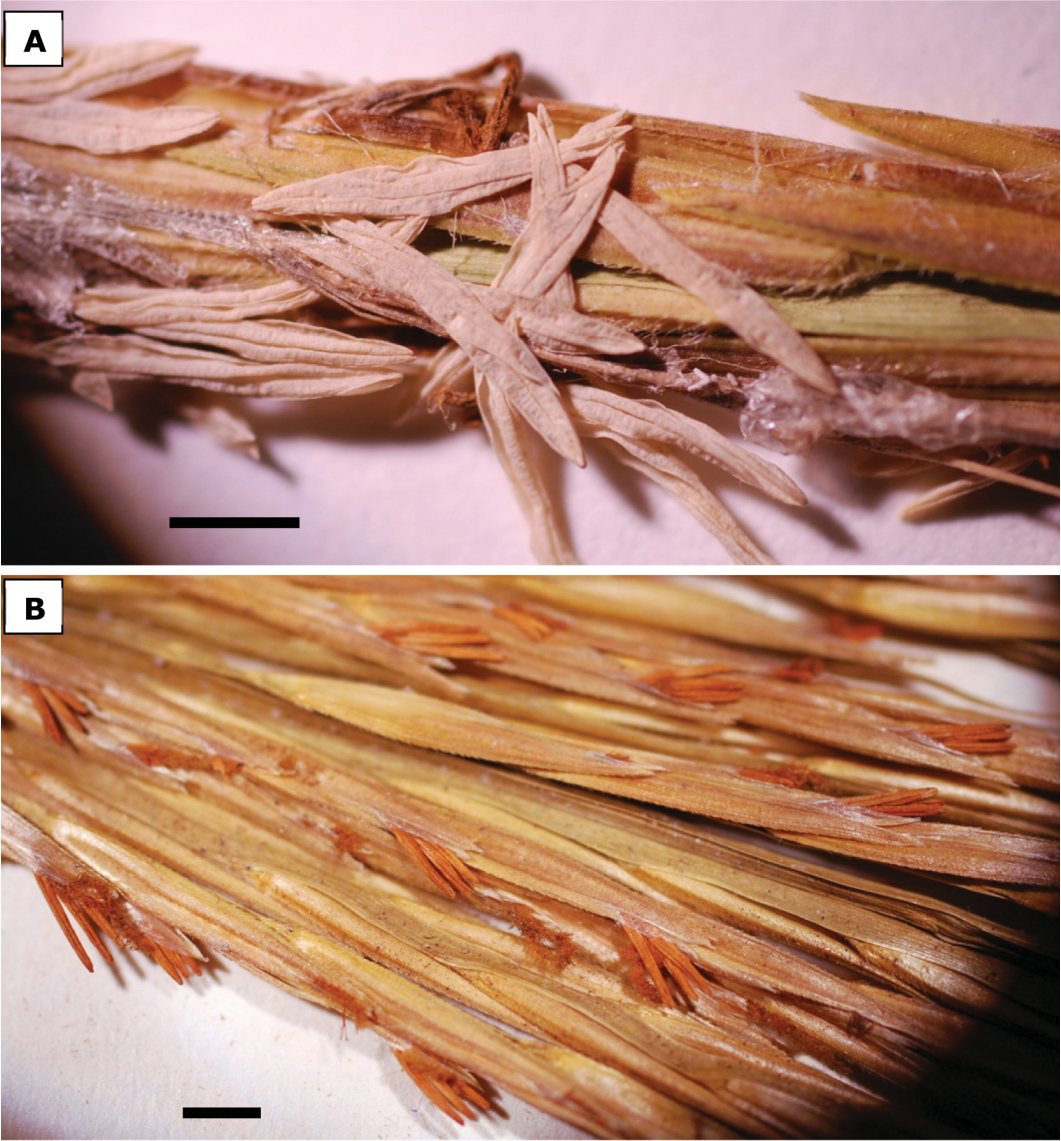
Anthers of **a** *Spartina anglica* [U.S.A., Washington, Pacific Co., Zika 17595, WTU], bar = 3 mm. **b** *Spartina ×townsendii* [England, Hythe, Southampton, Marchant s.n., UBCV221074], bar = 3 mm. Anthers of *Spartina anglica* are fully exserted at anthesis, dehiscent, and the pollen is fertile. The longitudinal splitting of the anthers is a good indicator of dehiscence. Anthers in *Spartina ×townsendii* are not or incompletely exserted at anthesis, indehiscent, and the pollen is sterile. Photos: J.M. Saarela.

**Figure 5.**
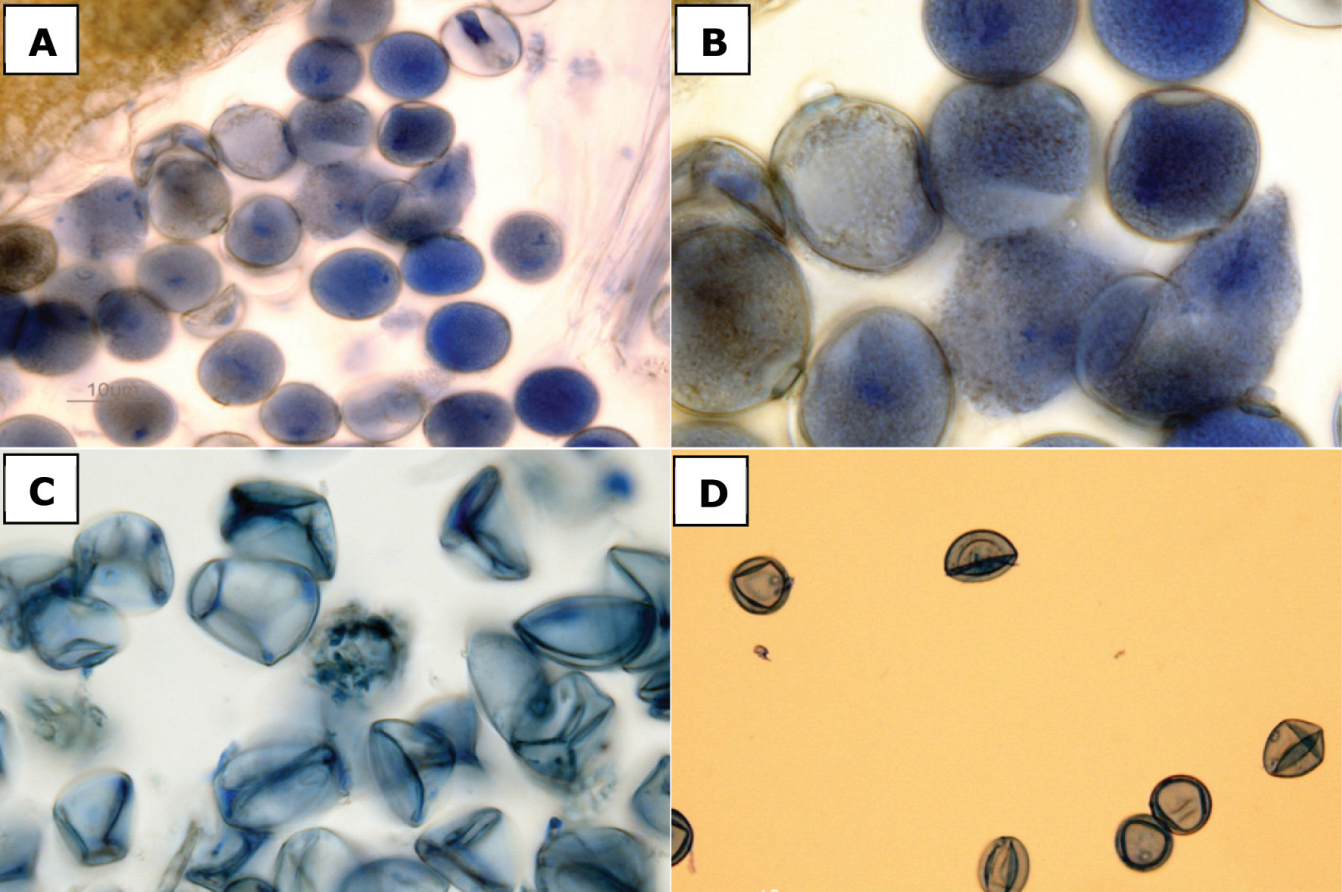
Pollen stained with lactophenol cotton blue. **a, b** *Spartina anglica*, fertile pollen [Canada, British Columbia, Williams 2004-3 (CAN)] **c** *Spartina ×townsendii*, sterile pollen [England, Hampshire, 1877, Groves s.n. (CAN)] **d** *Spartina ×townsendii*, sterile pollen [Canada, British Columbia, Saarela & Percy 791 (CAN)]

Overall, plants of *Spartina anglica* tend to be larger than those of *Spartina ×townsendii*, including the lengths of reproductive structures useful in distinguishing the taxa. The species can be distinguished with careful measurements on herbarium specimens, though there is some overlap in the diagnostic morphological characteristics. When making a determination, multiple characters should be examined and multiple measurements should be made on a single plant when possible. *Spartina anglica* is distinguished from *Spartina ×townsendii* by its longer spikelets [(15–)16.5–25 mm long vs. 14–17.5 mm long]; longer anthers [7–10 mm long vs. 5–7(–8.5) mm long]; anthers that are fully exserted and dehiscent [vs. anthers that are not or incompletely exserted and indehiscent; Fig. 4]; fertile pollen [vs. sterile pollen (see below and Fig. 5)]; longer ligules [1–3 mm long vs. 1–1.5 mm long]; 3–6-veined upper glumes [vs. 3-veined upper glumes]; and longer upper glumes [13–22 mm long vs. 12.5–16.5 mm long]. Marchant (1968a) observed that leaf blade angle with respect to the culm can be used to differentiate the taxa, as the blades tend to be more erect in *Spartina ×townsendii* [blades forming an angle 30–40° with respect to the culm] and more spreading in S. *anglica* [blades forming an angle of 30–60°with respect to the culm], and this character is noted in many of the recent field identification resources for the Pacific Northwest. Leaf angle is sometimes difficult to determine on herbarium specimens. Marchant (1968a) also noted swards of *Spartina anglica* in the field (in England) differed from swards of *Spartina ×townsendii* as being less dense in tillers (ca. 52/100 cm^2 ^vs. ca. 96/100 cm^2^) and having more robust but fewer rhizomes.

*Spartina anglica* can be distinguished from *Spartina alterniflora* by its longer spikelets [(15–)16.5–25 mm long vs. 8–14(–16.5) mm long], moderately to densely pubescent glumes (vs. glabrous or weakly pubescent glumes) and its longer anthers (7–10 mm long vs. 3–6 mm long). *Spartina anglica* is readily distinguished from *Spartina densiflora*, *Spartina gracilis*, *Spartina patens* and *Spartina pectinata* by its glabrous leaf blade margins [vs. scabrous leaf blade margins].

#### Pollen fertility.

Determining pollen fertility by staining anthers with lactophenol cotton blue is a useful way to distinguish male sterile hybrid plants from those that are fertile, as the cytoplasm of fertile pollen grains readily takes up the stain whereas sterile (i.e., aborted) pollen grains do not. Pollen staining is thus an effective, though more technically involved method, to definitively distinguish the fertile *Spartina anglica* from the sterile F1 hybrid *Spartina ×townsendii*, as demonstrated by Marchant (1968a). To confirm identifications of these taxa here, and to demonstrate the utility of this technique, I determined pollen fertility for multiple herbarium specimens of *Spartina anglica* and *Spartina ×townsendii* using lactophenol cotton blue; collections examined in this manner are identified with the symbol † in the specimen list below and under *Spartina ×townsendii*. One to three anthers were sampled from a spikelet on each specimen, and these were macerated with forceps in a drop of water on a glass slide to release the pollen. One or a few drops of lactophenol cotton blue were added to the slide and after sitting for a minimum of ten minutes, the slides were examined at 20× to 60× magnification with a compound microscope. In general, the number of pollen grains recovered per specimen varied with the stage of pollen development. For example, in specimens of *Spartina anglica* that had been collected during or after dehiscence, fewer pollen grains were available for study, whereas in *Spartina ×townsendii* dehiscence does not occur and abundant pollen grains were recovered from the anthers. In fertile pollen grains the cytoplasm was evidently visible and stained, whereas in sterile pollen there was no or little evidence of cytoplasmic staining (Fig. 5). All plants that were determined morphologically to be *Spartina anglica* had fertile pollen, and all plants determined as *Spartina ×townsendii* had sterile pollen.

#### Specimens examined.

**Canada. British Columbia:** Greater Vancouver Regional District: Lower Fraser Valley, Boundary Bay, foot of 104^th^ Street, 49°02'00"N, 122°56'00"W, 26 May 2004, P.Lim s.n. (V [V191319]); Lower Fraser Valley, Boundary Bay, foot of 112^th^ Street, 49°02'00"N, 122°56'00"W, 16 Jun 2004, P.Lim s.n. (V [V191320]]); Delta, Boundary Bay Regional Park, off 12^th^ Avenue, 49°00'N, 123°02'W, 30 Jul 2004, G.Williams 2004-3 (UBC [UBCV220132†, Suppl. Fig. 4], V [V191495]); Delta, 50 m E of Beach Grove dike, Boundary Bay, 49°02'N, 123°03'W, 30 July 2004, G.Williams 2004-2 (CAN [CAN592131†, Suppl. Fig. 5], UBC [UBCV220131†, Fig. 3], V [V191494]); Robert Banks Causeway, S shore, 49°05'N, 123°11'W, 30 Jul 2004, G.Williams 2004-1 (UBC [UBCV220130†, Suppl. Fig. 6], V [V191493]). **United States of America. Washington:** Island Co.: W side of Cornet Bay mudflat N of group camp in Deception Pass State Park, NE ¼ of NE ¼ of Sec. 35, T.34N, R.1 E., 7 Aug 2008, J.Walker 382 (WTU [WTU373426†, Suppl. Fig. 7], Suppl. Fig. 7); Puget Sound, Whidbey Island, N shore of Kennedy’s Lagoon, E side of Route 525, SW of S end of Zylstra Road, 48°14'N, 122°43.9'W, 22 Aug 2002, P.F.Zika 17595 (WTU [WTU365225†, Suppl. Fig. 8); Livingston Bay, Iverson Spit, 48°13'26"N, 122°26'19"W, 20 July 1983, R.E.Frenkel 3045 (UBC [UBCV196071†, Suppl. Fig. 9], WTU [WTU305390†, Suppl. Fig. 10]). Jefferson Co.: intertidal zone on the N side of the mouth of Chimacum Creek on the Quimper Peninsula, T29N R1W S35, 2 Aug 2004, F.Weinmann & A.Weinmann 233 (WTU [WTU370619†, Suppl. Fig. 11]). Snohomish Co.: island at W end/mouth of Ebey’s Slough, 48°02.555'N, 122°12.535'W, 15 Sep 2005, D.Giblin & B.Legler 244 (WTU [WTU364297†], Suppl. Fig. 12); mouth of Hat Slough, S side, 48°11.782'N, 122°22.463'W, 23 Sep 2005, D.Giblin & B.Legler 270 (WTU [WTU364317†], Suppl. Fig. 13); Puget Sound, Camano Island, E side of Davis Slough, N side of Route 532, 1.5 air km W of Stanwood, 48°14.4'N, 122°23.3'W, P.F.Zika 19170 (WTU [WTU355103†], Suppl. Fig. 14). **Denmark.** Jutland,NE coast of Rømø, 55°08'N, 08°31'E, 25 Aug 1970, I.B.Jørgensen & J.Svendsen 368 (CAN [CAN358861†, Suppl. Fig. 15]). **England.** England, 1879, H.Groves & J.Groves s.n. (US [US747577]); Poole Harbour, Fitzworth Point, R.O.Sherring s.n. (US [US1271872]). Devon Co.: South Devon, Combeinteignhead, between Newton Abbot and Teignmouth, ca. 50°32'N, 03°32'W, 24 Aug 1979, G.A.Matthews s.n. (CAN [CAN448159†, Suppl. Fig. 16]). Dorset Co.: Poole, 50°43'N, 01°59'W, Oct 1905, W.H.J.Riddelsdell 1734 (CAN [CAN467908†, Suppl. Fig. 17]); Dorset, E of Poole Harbour, 50.6863°N, 2.0181°W uncertainty 2195 m, Sep 1910, H.S.Thompson s.n. (UBC [UBCV1679, Suppl. Fig. 18]). Hampshire Co.: Hayling Island, Hants, 50.7999°N, 0.9667°W uncertainty 7194 m, 20 July 1938, T.M.C.Taylor 5378 (UBC [UBCV69243, Suppl. Fig. 19, UBCV20934, Suppl. Fig. 20]); between the “Sturt” Pond, [illegible], and Hurst Castle, South Hants, Sep 1910, J.C.Melvill 1841 (UBC [UBCV1678, Suppl. Fig. 21]); Lymington, towards Lymington Spit almost one mile from land, G.Stapf s.n. (US [US1271838]); Hythe, S of town and near road and Admiraly buildings, 28 Jun 1961, C.E.Hubbard 286961 (US [US3055889]). Isle of Wight, near Quarr Abbey, Oct 1907, O.Stapf (US [US1271833]). **FRANCE.** Baie de Mont Saint Michel, Sep 1933, P.de la Varde s.n. (US [US1611439]); mouth of river at Saranelle, near Cannes, 43°33'05"N; 07°00'45"W, Oliver s.n. (CAN [CAN421006†, Suppl. Fig. 22]).

### Spartina densiflora

Brongn., Voy Monde 2(2): 14. 1829.

http://species-id.net/wiki/Spartina_densiflora

Spartina densiflora Brongn., Voy Monde 2(2): 14. 1829. **Type**. CHILE. D’Urville s.n. (holotype: P [P02243277!]; isotype: US [US92018!] (fragm. ex P & photo)).
Chauvinia chilensis Steud., Syn. Pl. Glumac 1: 362. 1854. **Type.** CHILE. Concepcion, J.S.C.D. D’Urville s.n. (holotype: P [P02243276!]; isotype: US [US865687A!] (fragm. ex P)).

#### Description.

Culms to 96 cm tall, cespitose from hard knotty bases, rarely with short rhizomes, forming dense tufts. Sheaths glabrous, often purple-tinged; ligules 1–2 mm long; blades to 32 cm long × 1–2 mm wide, involute for most or all of their length, wider proximally when flat, adaxial surfaces scabrous, abaxial surfaces glabrous, margins scabrous. Inflorescences 10.5–17 cm long × 6–8(–10) mm wide at midpoint, with (2–)6–9(–15) branches; branches 3–6(–7.5) cm long × 2–3 mm wide, appressed, conspicuously decreasing in length towards inflorescence apex, rachises 0.8–1 mm wide between spikelets, not extending beyond terminal spikelet, glabrous, margins glabrous or scabrous. Spikelets 9–13 mm long × 1.5–2 mm wide, tightly appressed, strongly overlapping; calluses 1–1.5 mm long. Glumes glabrous or scabrous, when present hairs < 0.1 mm long, keels scabrous, teeth 0.1–0.2 mm long, margins usually purple-tinged; lower glumes 4–7 mm long × 0.5–0.7 mm wide, 1-veined; upper glumes 7.5–11.5 mm long × 1–1.5 mm wide, 1-veined; lemmas 6–9 mm long, glabrous or minutely scabrous, keels scabrous distally, glabrous proximally; paleas exceeding lemmas by 0.5 mm, glabrous; anthers 3–4 mm long, yellowish, exserted at maturity, pollen fertile. 2*n* = 70 (Ayres et al. 2008, Fortune et al. 2008)

#### Common name.

Austral cordgrass.

#### Etymology.

The epithet *densiflora* refers to the densely-flowered inflorescences of the species.

#### Illustration.

Barkworth 2003:251.

#### Distribution.

Native to South America in temperate coastal regions of southern Brazil, Uruguay, Argentina, and on the coast of Chile (Bortolus 2006). Introduced in Spain, Portugal, the Mediterranean (see Castillo et al. 2000, Nieva et al. 2005), United States (California, Washington), and Canada (British Columbia).

#### Comments.

The taxonomy, biogeography, and natural history of *Spartina densiflora* is reviewed by Bortolus (2006). The synonymy presented here is not complete; several taxa described from South America are considered to be synonyms by Mobberley (1956), including *Spartina montevidensis* Arech. (see Carrasco et al. 2002 for the location of a probable type specimen), *Spartina patagonica* Speg., and several taxa described by St-Yves (1932). A modern taxonomic revision is needed for *Spartina densiflora* to clarify its taxonomy and morphological variation throughout its range (Bortolus 2006), particularly given its rapidly increasing global footprint.

In North America, *Spartina densiflora* is present in California, Washington, and British Columbia. It was first established in California. It occurs in Humboldt Bay, where it is thought to have been introduced by shipping in the late 1800s (Spicher and Josselyn 1985, Kittleson and Boyd 1997). Prior to the early 1980s, this population was erroneously thought to be a native form of *Spartina foliosa* (Bortolus 2008), and seed collected from plants growing in Humboldt Bay was introduced to San Francisco Bay (see Faber 2000), and independently to Richardson Bay. Hybrids between *Spartina densiflora* and *Spartina foliosa* have been confirmed recently from San Francisco Bay in a region where both parental species are present (Ayres et al. 2008). These are not well characterized morphologically in the literature.

*Spartina densiflora* was documented in Washington a decade ago. The first collection was made in 2001 on Whidbey Island at the northern boundary of Puget Sound (Heimer 01-1 WTU, UBC). Caplow (2002) reported a 1/10 acre infestation of *Spartina densiflora* at the west end of Grays Harbor (46.9408°N, 124.04583°W) in the southeastern part of the state. By 2006 this infestation had expanded substantially, covering some 10 acres (Murphy et al. 2007). Kozloff (2005) included *Spartina densiflora* in his *Spartina* treatment, noting only ‘coastal salt marshes’ as its distribution.

**Figure 6.**
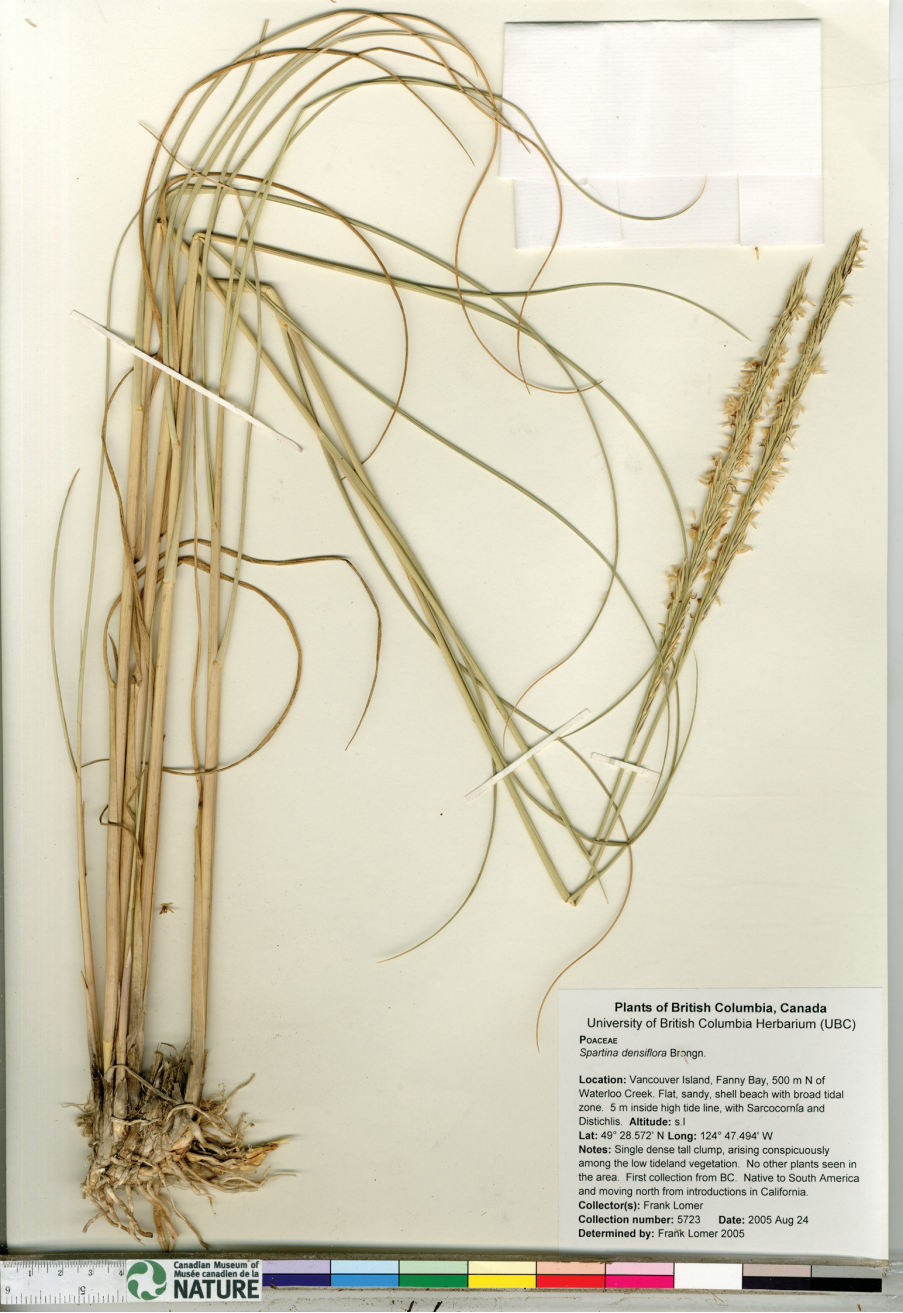
Photograph of a specimen of *Spartina densiflora* collected at Fanny Bay, Vancouver Island, British Columbia (Lomer 5723, UBC). Image published with the permission of the University of British Columbia Herbarium, Beaty Biodiversity Museum.

*Spartina densiflora* is now invading British Columbia, where it was first found in 2005 in Bayne’s Sound, a channel between Vancouver Island and Denman Island (Morgan and Sytsma 2010). It is not reported for the province in several recent floras that consider the region (Douglas et al. 2001, Barkworth 2003, Kozloff 2005), but is included in the online E-Flora BC (Anonymous 2011). In the Bayne’s Sound region, only a few herbarium collections documenting the species have been made; these are from Fanny Bay (Lomer 5723, CAN, Fig. 6) and the Buckley Bay Ferry Terminal (Lomer 7377, CAN). Some data on the extent of the invasion are given by Morgan and Sytsma (2010) and Dresen et al. (2010), who mapped the species in Bayne’s Sound where the extent of its local distribution ranges from individual plants to multiple five meter patches. Detailed characterization of this invasion has not otherwise appeared in the literature.

#### Morphology.

The description presented here is based on the few specimens that have been collected in Washington and British Columbia and deposited in herbaria (see Specimens Examined). Variation in some characters, particularly vegetative characteristics such as leaf length and culm height, is probably greater than recorded here. For example, Mobberley (1956) reported that culms in the species range to 1.5 m tall. For a description based on material from the native range of *Spartina densiflora*, see Mobberley (1956).

**Figure 7.**
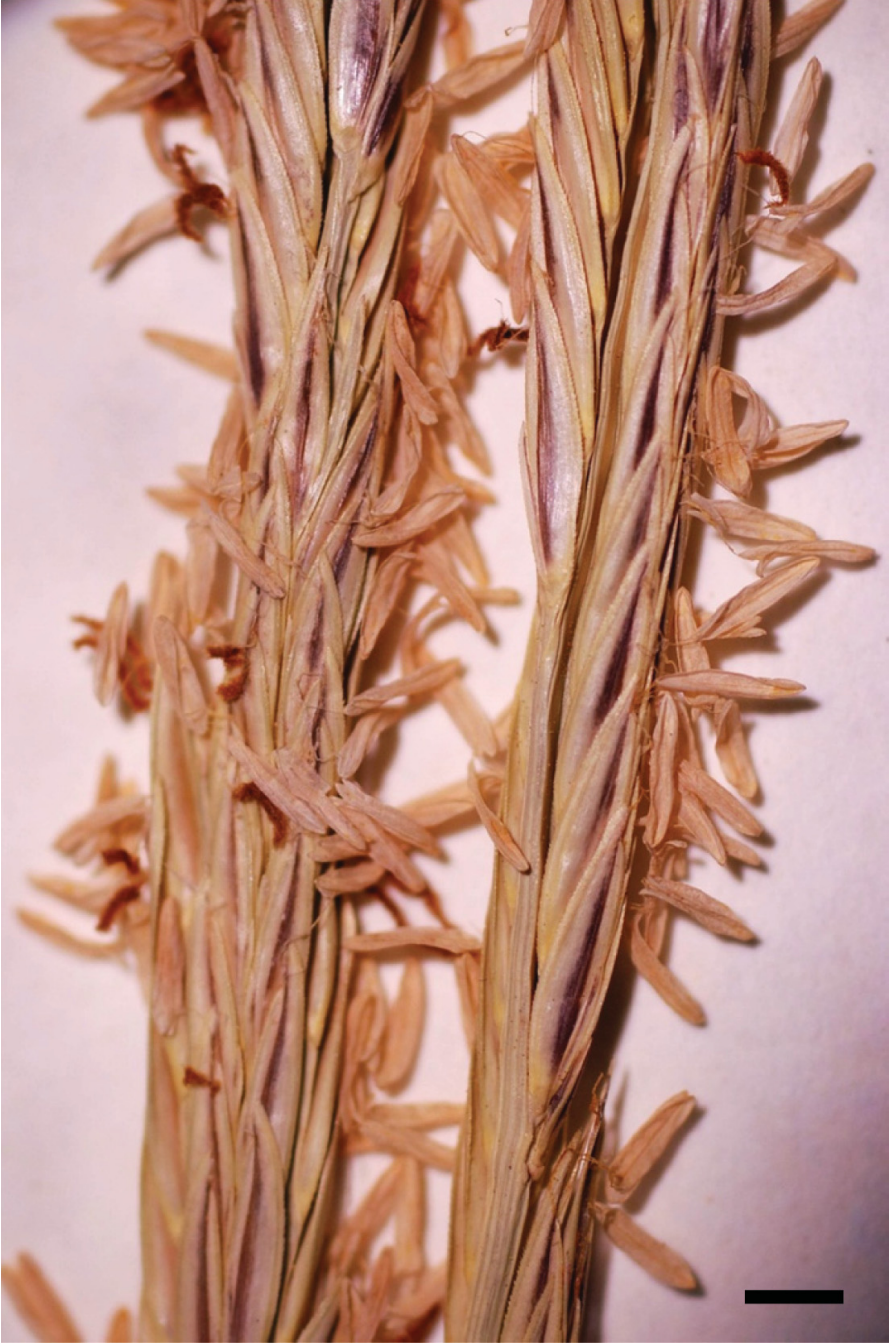
Spikelets of *Spartina densiflora* (Canada, British Columbia, Lomer 5723, UBC). Bar = 3 mm. Photo: J.M. Saarela.

*Spartina densiflora* can be distinguished from *Spartina alterniflora*, *Spartina anglica*, and *Spartina ×townsendii* by the following combination of characters: plants cespitose [vs. strongly rhizomatous]; blades involute for all or most of their length [vs. blades flat proximally, involute distally]; blade margins scabrous [vs. blade margins smooth]; branch rachises not prolonged beyond the terminal spikelet [vs. branch rachises prolonged beyond the terminal spikelet as a bristle, rarely not prolonged]; glume margins often purple-tinged [vs. glume margins whitish, not purple-tinged] (Fig. 7); and spikelets tightly appressed and strongly overlapping, concealing the rachis between spikelets [vs. spikelets weakly appressed and weakly to moderately overlapping, with portions of the rachis usually visible between spikelets]. In the field, the leaf margins of *Spartina densiflora* may feel smooth to the touch, as the involute blades often conceal the leaf margins where the scabrous teeth are located; these scabrous teeth are best observed with a microscope (F. Lomer, personal communication, 2012). *Spartina densiflora* is readily distinguished from *Spartina gracilis*, *Spartina patens*, and *Spartina pectinata* by its cespitose habit [vs. rhizomatous].

#### Specimens examined.

**Canada. British Columbia:** Vancouver Island, Fanny Bay, 500 m N of Waterloo Creek, 49°28.572'N, 124°47.494'W, 24 August 2005, F.Lomer 5723 (UBC, Fig. 6); Vancouver Island, 20 km south of Courtenay, Buckley Bay Ferry Terminal, north side of dock at high tide level, single clump, sea level, 49°31'32"N, 124°50'54.5"W, 12 July 2010, F.Lomer 7377 (CAN, Suppl. Fig. 23). **United States of America. Washington:** Island Co.: Whidbey Island, near Coupeville (T32E R2E S37), 14 Nov 2001, D.Heimer 01-01 (UBC [UBCV224048, Suppl. Fig. 24], WTU [WTUb349303, Suppl. Fig. 25]).

### Spartina gracilis

Trin.

http://species-id.net/wiki/Spartina_gracilis

Spartina gracilis Trin.**,** Mém. Acad. Imp. Sci. Saint-Pétersbourg, Sér. 6, Sci. Math., Seconde Pt. Sci. Nat. 6,4(1-2): 110. 1845. **Type**. CANADA.Amer. bor., Hooker s.n. (isotypes: LE [LETRIN2058.01!], US [US92014!] (fragm. ex LE)).

#### Description:

See Mobberley (1956), Barkworth (2003), and Douglas et al. (2001). 2*n* = 40 (Marchant 1968b, Reeder 1977).

#### Common name.

Alkali cordgrass, big cordgrass.

#### Illustrations.

Hitchcock 1935:493, Hitchcock 1951: 513, Hitchcock et al. 1969:706, Barkworth 2003:251, Douglas et al. 2001:269.

#### Etymology.

The Latin epithet *gracilis* means “thin, slender” (Stearn 1992), probably given in reference to the general habit of the species.

#### Distribution.

Southern Northwest Territories, Canada, to central Mexico (Barkworth 2003). In the Pacific Northwest *Spartina gracilis* is distributed in the steppe and montane zones of the south central and south eastern parts of British Columbia (Douglas et al. 2001), and in eastern Oregon and Washington (Barkworth 2003, Wilson et al. 2012). It is not a salt marsh species and is not invasive.

#### Morphology.

**Figure 8.**
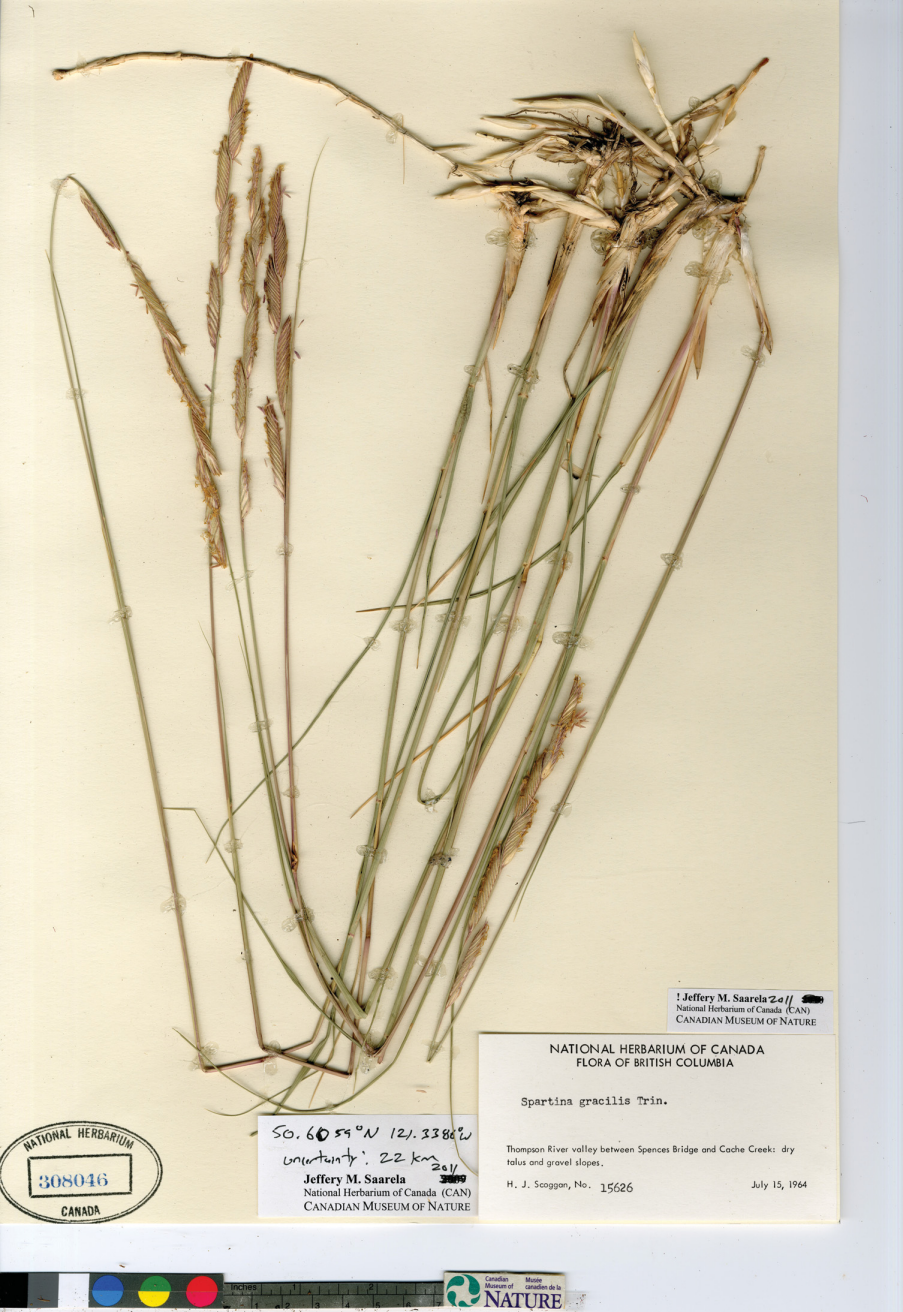
Photograph of a specimen of *Spartina gracilis* collected in the Thompson River Valley, British Columbia (Scoggan 15626, CAN).

*Spartina gracilis* is a distinctive taxon (Fig. 8). In the Pacific Northwest it is likely to be most readily confused with *Spartina pectinata*, which also grows inland. It is distinguished from *Spartina pectinata* by the following combination of characters: upper glumes unawned or short-awned, awns to 2 mm long [vs. distinctly awned upper glumes, awns 3–8 mm long], ciliate glume keels [vs. pectinate glume keels], spikelets 6–11 mm long [vs. 10–25 mm long], ligules 0.5–1 mm long [vs. 1–3 mm long], 3–12 branches per inflorescence [vs. 5–50 branches per inflorescence], and 10–30 spikelets per branch [vs. 10–80 spikelets per branch]. It is distinguished from *Spartina patens* by its ciliate glume keels [vs. scabrous glume keels], inconspicuous lateral veins on the upper glumes [vs. conspicuous lateral veins on the upper glumes], most branches 3–6 mm wide [vs. most branches 2–3 mm wide], branches closely appressed to the main axis [vs. branches appressed, ascending or spreading from main axis], and florets more or less equaling the upper glumes in length [vs. florets shorter than the upper glumes]. *Spartina gracilis* can be readily separated from *Spartina alterniflora*, *Spartina anglica* and *Spartina ×townsendii* by its scabrous leaf margins [vs. glabrous leaf margins], and from *Spartina densiflora* by its rhizomatous habit [vs. cespitose].

#### Specimens examined.

**CANADA. British Columbia:** 23 mi W of Kamloops, 50.6667°N, 120.8569°W uncertainty 33215 m, 23 July 1941, W.A.Weber 2548 (CAN [CAN33940]); Thompson River valley between Spences Bridge and Cache Creek, 50.6059°N, 121.3386°W uncertainty 22 km, 15 July 1964, H.J.Scoggan 15626 (CAN [CAN308046], Fig. 8); Flying U Ranch, Cariboo, bank at edge of Green Lake, 51.4172°N, 121.2025°W uncertainty 8569 m, 21 June 1944, J.W.Eastham 11509 (CAN [CAN33941]); N of Kamloops, 50.6667°N, 120.3333°W uncertainty 7196 m, 13 Jun 1889, J.Macoun s.n. (CAN [CAN33943, Suppl. Fig. 26]); Kamloops, 50.6667°N, 120.3333°W uncertainty 7196 m, 4–7 Sep 1931, V.Kujala & A.Cajander s.n. (CAN [CAN394081, CAN394014, Suppl. Fig. 27]); 119 mile, Cariboo, 18 Jun 1942, J.A.Munro 23 (CAN [CAN33942]); Similkameen River, 10 Jun 1905, J.M.Macoun 77227 (CAN [CAN33944, Suppl. Fig. 28]). **UNITED STATES OF AMERICA. Montana:** Hound Creek, 30 Jul 1883, F. Lamson-Scribner 329 (CAN [CAN162196]). **North Dakota:** Ward County, 26 Jul 1963, L.F.Lautenschlager 694 (CAN [CAN297003]). **Utah:** Death Ridge-Horse Mountain Road, near Caanan Peak, 6800 ft, 15 June 1990, M.A.Franklin & D.Atwood 7129 (CAN[CAN563733]). **Washington:** Okanogan Co.: Loomiston, Aug 1897, A.D.E.Elmer 891 (CAN [CAN162194, CAN162191]).

### Spartina patens

(Aiton) Muhl., Descr. Gram. 55. 1817.

http://species-id.net/wiki/Spartina_patens

Spartina juncea var. *patens* (Aiton) St.-Yves, Candollea 5: 86. 1932. *Dactylis patens* Aiton, Hort. Kew. 1: 104. 1789. **Type**. United Kingdom. Cultivated, native of North America, introduced 1781, by Mr. William Curtis (holotype: BM-001010621!).
Trachynotia juncea Michx., Fl. Bor.-Amer. 1: 64. 1803. *Limnetis juncea* (Michx.) Rich., Syn. Pl. 1: 72. 1805. *Spartina juncea *(Michx.) Willd., Enum. Pl. 81. 1809. *Spartina patens* var. *juncea* (Michx.) Hitchc., Rhodora 8: 210. 1906. *Spartina juncea* subvar. *americana* St.-Yves, Candollea 5: 84. 1932. **Type.** United States of America. bord des Creeks salés Basse Caroline, Michaux s.n. (holotype: P, not seen). Note: See Hitchcock (1908:153) for notes on the type specimen. Treatment of *Trachynotia juncea* as a synonym of *Spartina patens* follows Mobberley (1956).
Limnetis juncea  var. *monogyna* M.A.Curtis, Boston J. Nat. Hist. 1: 136. 1837. *Spartina patens* var. *monogyna* (M.A.Curtis) Fernald, Rhodora 49: 114. 1947. **Type.** United States of America. North Carolina: M.A.Curtis s.n. (holotype: GH [GH00295532!], Appendix Fig. 3). Note: Fernald (1947:115) referred to this specimen as “The isotype of Curtis’s variety in the Gray Herbarium…” and “The Curtis type or isotype ….”; it likely is the holotype.
Spartina pumila Roth, Catal. Bot. 3: 10. 1806. **Type.** United States of America. New York, 1794, Schreber s.n. (holotype: B-Willd.!).

#### Description.

See Mobberley (1956), Barkworth (2003), Douglas et al. (2001), and Lonard et al. (2009). 2*n* = 40 (Marchant 1968b).

#### Common names.

Saltmeadow cordgrass, saltmeadow grass, marsh hay, wiregrass, foxgrass, couchgrass, rush saltgrass, spartine étalée (Lonard et al. 2010).

#### Etymology.

The Latin epithet *patens* means “spreading, outspread” (Stearn 1992), given in reference to the general habit of the species, which forms dense, monotypic stands.

#### Illustrations.

Hitchcock 1935: 494; Hitchcock 1951:514; Holmgren et al. 1998:750, Douglas et al. 2001:271, Barkworth 2003:251.

#### Distribution.

Native to the east coast of North America and Central America, distributed along the Atlantic coast of Newfoundland and Labrador to Texas, the Atlantic coast of Mexico, and throughout the Caribbean Islands (e.g., Barkworth 2003, Beetle 1977, Howard et al. 1989, Liogier and Martorell 2000, Peterson 2001). It has been introduced to coastal regions in Spain (Campos et al. 2004, SanLeón et al. 1999, Prieto et al. 2011), China (An et al. 2007), North Africa, and the west coast of the United States (Oregon, Washington), and Canada (British Columbia).

#### Comments.

*Spartina patens* grows in coastal salt marshes and brackish waters, where it usually forms dense stands above the intertidal zone and into higher and drier areas of the salt marsh (Mobberley 1956). In Europe this taxon is sometimes treated as a separate species, *Spartina versicolor* Fabre, a traditional circumscription that some contemporary authors maintain (e.g., Sánchez Gullón 2001; Clayton et al. 2002 onwards; see SanLeón et al. 1999 and Prieto et al. 2011 for summaries of this taxonomic problem). In North America *Spartina patens* hybridizes with *Spartina pectinata* in areas where they grow together, forming the hybrid taxon *Spartina ×cespitosa*. This hybrid is known from scattered locations along the Atlantic coast from Prince Edward Island to Maryland (Mobberley 1956, Dore and Marchant 1968, Barkworth 2003), but has not been recorded along the Pacific coast. The biology of *Spartina patens* is reviewed by Lonard et al. (2010).

In Oregon, *Spartina patens* grows in the Siuslaw estuary on Cox Island (Lane Co.), where it has been introduced since at least 1939 and has expanded considerably since that time (Frenkel and Boss 1988). It is not reported from elsewhere in Oregon (Wilson et al. 2012). In Washington it has been reported from Dosewallips State Park on the west shore of Hood Canal (Frenkel 1987). In California it is present in Southampton Marsh in San Francisco Bay (Spicher and Josselyn 1985, Baird and Thieret 2012).

**Figure 9.**
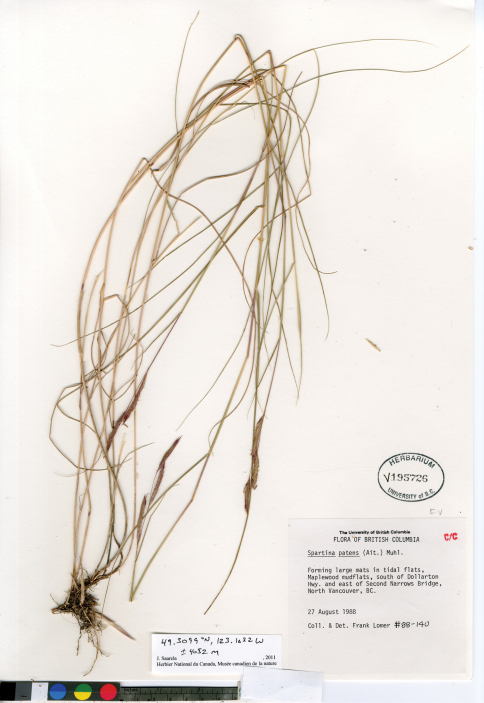
Photograph of a specimen of *Spartina patens* collected at Maplewood mudflats, North Vancouver, British Columbia (Lomer 88–140, UBC). Image published with the permission of the University of British Columbia Herbarium, Beaty Biodiversity Museum.

*Spartina patens* was the first of the invasive cordgrasses to be collected in British Columbia. It was discovered in 1979 in the Comox Estuary on Vancouver Island (Brayshaw 79-1143, V); nearly a decade later, in 1988, it was collected on the adjacent mainland coast in Burrard Inlet, North Vancouver (Lomer 88–140, UBC, Fig. 9). *Spartina patens* was recognized as part of the provincial flora by Douglas et al. (1994) based on collections from these two localities, and is reported for the region in recent floras (Barkworth 2003, Douglas et al. 2001). Large stands of the species were reported in 2000 at the Comox locality in the Courtenay River estuary at the delta of Brooklyn Creek to the embayed area of Goose Spit (Asp and Adams 2000). *Spartina patens* is also reported from Port Moody Arm of Burrard Inlet in British Columbia (Dresen et al. 2010). Voucher specimens from these latter two sites should be collected and deposited in herbaria.

#### Morphology.

*Spartina patens* exhibits considerable morphological variation and several authors have recognized two infraspecific taxa (see Mobberley 1956). Mobberley (1956) studied this variation across the native range of the taxon and found plants at the northern and southern extremes of the taxon’s range to be distinct, but considerable overlap and clinal variation from north to south in areas between the extremes. Based on these data he argued against the recognition of infraspecific taxa in *Spartina patens*, a circumscription followed by subsequent authors.

Based on specimens of *Spartina patens* examined here collected in Oregon, Washington, and British Columbia, introduced plants in these areas are similar morphologically to those in the eastern Canada and the northeastern United States, which tend to be smaller than plants distributed further south (Mobberley 1956).

*Spartina patens* can be distinguished from *Spartina densiflora* by the following combination of characters: branches distinctly one-sided, appressed, ascending or spreading from main axis, distant or weakly overlapping, and approximately the same length within an inflorescence [vs. branches not distinctly one-sided, appressed, strongly overlapping, and conspicuously decreasing in length towards the inflorescence apex], rhizomes wiry, plants forming dense mats [vs. rhizomes absent, rarely short, plants cespitose, forming distinct clumps], upper glumes distinctly 3-veined [vs. upper glumes 1-veined], and ligules 0.5–1 mm long [vs. ligules 1–2 mm long].

*Spartina patens* can be distinguished from *Spartina alterniflora*, *Spartina anglica* and *Spartina ×townsendii* by: blade margins and adaxial surfaces scabrous [vs. blade margins and adaxial surfaces glabrous], blades 0.5–4 mm wide at base, involute for most or all of their length [vs. blades 3–10 mm wide at base, often involute distally], branches distinctly one-sided, distant or weakly overlapping [vs. branches not distinctly one sided, strongly overlapping], rhizomes thin and wiry [vs. rhizomes thick and fleshy], upper glumes conspicuously 3-veined [vs. upper glumes 1–3-veined, veins inconspicuous], and spikelets usually purple-tinged [vs. spikelets rarely or never purple-tinged].

#### Specimens examined.

**Canada. British Columbia:** Vancouver Island, Goose Spit, Comox, 49°40'N, 124°54'W, 14 Sep 1979, T.C.Brayshaw 79-1143 (V [V95308]); Vancouver Island, based of Comox Spit, 49°40'N, 124°54'W, 7 Aug 1984, T.C.Brayshaw 84-139 (V [V127117]); N shore of Burrard Inlet, E of Second Narrows Bridge, North Vancouver, 49.3099°N, 123.1032°W uncertainty 4052 m, sea level, Jul 1987, F.Lomer 87-001 (UBC [UBCV194265, Suppl. Fig. 29]); North Vancouver, Maplewood Mudflats, S of Dollarton Highway and E of Second Narrows bridge, 49.3042°N, 123.0009°W uncertainty 500 m, 27 Aug 1988, F. Lomer 88-140 (UBC [UBCV195726, Fig. 9); North Vancouver, Maplewood mud flats, 49.3042°N, 123.0009°W uncertainty 500 m, 16 Sep 1993, F.Lomer s.n. (V [V169504, V169505]). **New Brunswick:** St. Andrew’s, 16 Aug 1900, J.Fowler s.n. (CAN [CAN390994]). Charlotte Co.: Grand Manan, 44°41'53"N, 66°49'20"W, 31 Jul 1944, C.A.Weatherby & U.F.Weatherby 7300 (CAN [CAN33948]). Westmorland Co.: 1 mi E of Cape Bimet, 5 mi E of Shediac, 46°14'N, 64°27'W, 7 Aug 1981, M.Shchepanek & A.Dugal 3657 (CAN [CAN474807]); W of upper cape, 7 Aug 1964, P.R.Roberts & N.Bateman 64-2564 (CAN [CAN305984]); Memramcook, 45°58'15"N, 64°35'36"W, 21 Aug 1919, F.Rolland-Germaine 8022 (CAN [CAN332081]); Shediac, 46°13'17"N, 64°32'23"W, 5 Aug 1904, J.Fowler s.n. (CAN [CAN391711]); Moncton, 46°05'58"N, 64°47'59"W, 18 Sep 1912, M.O.Malte 108313 (CAN [CAN206830]). Restigouche Co.: Dalhousie, 48°2'55"N, 66°23'25"W, 4 Aug 1955, H.J.Scoggan 12683 (CAN [CAN240148]). **Newfoundland and Labrador:** Bonavista South District, Newman Sound Marsh, 48°32'15"N, 53°58'06"W, R.Charest, L.Brouillet, A.Bouchard & S.Hay 96-2065 (CAN [CAN58446]); St. George’s, 48°24'55"N, 58°29'40"W, 13 Aug 1910, M.L.Fernald & K.M.Wiegand 2597 (CAN [CAN33945]); St. George’s District, St. George’s, 48°24'55"N, 58°29'40"W, 4 Aug 1986, L.Brouillet & I.Saucier 86170 (CAN [CAN546790]; St. George’s District, Stephenville Crossing, saltmarsh NE of Main Gut bridge, 48°31'44"N, 58°27'19"W, 4 Aug 1986, L.Brouillet & I.Saucier 86183 (CAN [CAN546704]. **Nova Scotia:** N end of Summerville Beach, Summerville Center, 43°57'N, 64°49'W, 28 Sep 1979, D.F.Brunton & H.L.Dickson 2089 (CAN [CAN452656]; LeHave River, 6 Aug 1910, J.Macoun 82102 (CAN [CAN33949]. Cape Breton Co.: Grand Narrows, 45°57'24"N, 60°47'32"W, 27 Jul 1893, J.Macoun 21127, (CAN [CAN33952]); near mouth of George River, 27 Aug 1920, C.H.Bissell & D.H.Linder 19976 (CAN [CAN33951]. Digby Co.: Clare Municipality, Meteghan River, 44°13'N, 66°08'W, 30 Jul 1975, A.W.Dugal 75-131 (CAN [CAN475807]; Sissiboo River, Weymouth, 44°24'44"N, 65°59'43"W, 21 Aug 1920, M.L.Fernald, C.H.Bissell, C.B.Graves, B.Long & D.H.Linder 19974 (CAN [CAN33950]. Guysborough Co.: Canso, 45°20'12"N, 60°59'40"W, 15 Aug 1901, J.Fowler s.n. (CAN [CAN391709]. Hants Co.: mouth of Rennie Brook, East Walton, 17 Sep 1958, E.C.Smith, W.J.Curry, R.E.Clattenburg 18581 (CAN [CAN296579]). Kings Co.: Avonport, 45°06'01"N, 64°15'27"W, 23 July 1957, H.J.Scoggan 13850 (CAN [CAN255570]. Queens Co.: Port Mouton, 43°55'38"N, 64°50'55"W, C.H.Bissell & C.B.Graves 19978 (CAN [CAN33953]. Richmond Co.: Cape Breton Island, Fullers River Salt Marsh, 3 km W of Fourchu, off Hwy. 327, 45°43'N, 60°18'W, 23 Aug 1984, M.J.Shchepanek & A.W.Dugal 6426 (CAN [CAN521701]). Shelbourne Co.: Gunning Cove, 43°41'30"N, 65°20'45"W, 4 Oct 1982, S.J.Darbyshire 1790 (CAN [CAN487055]. Yarmouth Co.: Wedgeport, 43°44'23"N, 65°58'48"W 31 July 1953, W.L.Klawe 1278 (CAN [CAN298545]; Sand Beach, 43°48'43"N, 66°07'15"W, 7 Sep 1920, M.L.Fernald, B.Long, D.H.Linder 19977 (CAN [CAN33954]. **Ontario:** Essex Co.: Windsor, Windsor Salt Factory, 42°17'N, 83°06'W, 21 Sep 1975, P.M.Catling & S.M.McKay s.n. (CAN [CAN396523]); Windsor salt works, 24 Aug 1977, W.Botham 2011 (CAN [CAN459521], CAN [CAN459519]); Windsor, near salt factory, 29 Jul 1979, W.Botham 2182 (CAN [CAN459520]); Windsor, E side of Euclid Avenue bordering Detroit River, just S of Prospect Street, Ojibway Park, near salt plant, 42°17'N, 83°05'W, 3 Nov 1975, P.D.Pratt 18 (CAN [CAN440539]); Windsor, 50 m W of Prospect Avenue, along Euclid Road, E shore Detroit River, 42°17'N, 83°05'W, 4 Sep 1979, D.F.Brunton & P.D.Pratt 1915 (CAN [CAN452513]). **Prince Edward Island:** Prince Co.: Tignish, 46°57'02"N, 64°02'01"W, 6 Aug 1912, M.L.Fernald, B.Long & H.St-John 113172 (CAN [CAN33946]). Queens Co.: Long Creek salt marsh, ¼ mi E of Pond Point, 46°03'N, 62°57'W, 15 Aug 1981, M.Shchepanek & A.Dugal 4119 (CAN [CAN475274]); Brackley Point, 46°23'04"N, 63°11'06"W, 3 Aug 1888, J.Macoun 28967 (CAN [CAN33947]). **Quebec:** Maria Co.: Bonaventure, 48°03'N, 65°29'W, 11 Aug 1930, F.Marie-Victorin, F.Rolland-Germaine & E.Jacques 33799 (CAN [CAN33955]); Magdalen Islands, sandy sea strand at the Narrows, Alright Island, 21 Aug 1912, M.L.Fernald, B.Long & H.St-John 6880 (CAN [CAN33956]); Rivière-du-Loup Co.: Pointe-à-la-Loupe, L’Isle-Verte, 48°4'38"N, 69°16'28"W, 2 Sep 1957, E.Lepage 13956 (CAN [CAN252814]); Rivière-du-Loup, Hwy. 20, N shore of bay, 47°49'N, 69°32'W, 29 Sep 1979, H.L.Dickson & D.F.Brunton 3271 (CAN [CAN457594]). Kamouraska Co.: Rankin Point near Kamouraska, 24 Aug 1947, J.H.Soper & D.A.Fraser 3647 (CAN [CAN257674]); Rimouski Co.: Rimouski, 48°27'N, 68°32'W 30 Sep 1950, E.Lepage 13215 (CAN [CAN206869]); Le Bic, Cap aux Corbeaux, 48°23'N, 68°43'W, 30 Aug 1970, G.Lemieux 13630 (CAN [CAN444255]); L’Isle-Verte, 48°01'N, 69°20'W, 24 Aug 1951, L.McI.Terrill s.n. (CAN [CAN337522]); Tobin, 23 Aug 1951, L.McI.Terrill 6636 (CAN [CAN337521]); Iles-de-la-Madeleine, Cap de l’Est, 47°36'47"N, 61°27'35"W, s.d., M.M.Grandtner s.n. (CAN [CAN519307]); Dune du Nord, près de la Grande Lagune, 47°29'N, 61°45'W, 66.08.06, M.M.Grandtner 10697-V (CAN [CAN519268]); Ile-aux-Coudres, La Baleine, pointe E de l’île, 25 Aug 1977, J.Cayouette J77-133 (CAN [CAN466657]). **United State of America. Florida:** near Jacksonville, 17 Jul 1894, A.H.Curtiss 4948 (CAN [CAN373389]). **Louisiana:** Cameron Parish, along the Gulf of Mexico, S of an unnamed shell road which runs E from Cameron Parish Road 3106, on the E edge of Cameron, T15S, R9W, 30 Jun 1984, B.E.Button & D.W.Pritchett 2536 (CAN [CAN495018]); Jefferson Parish, roadside at Elmer’s Island, 2 Oct 1976, J.Guider 5023 (CAN [CAN432238]). **Maryland:** sea coast, Sep 1863, Wm.M.Canby s.n. (CAN [CAN162200]). **Massachusetts:** Barnstable Co.: Sandy Meck, Cape Cod, 41°44'00"N, 70°19'58"W, 28 Oct 1939, J.H.Soper 1109 (CAN [CAN257821, CAN316382]). Dukes Co.: Katama Bay, Edgartown, Marthas Vineyard, 41°21'15"N, 70°28'58"W, 11 Sep 1901, M.L.Fernald s.n. (CAN [CAN162201]). **New Jersey:** Atlantic City, 1880, C.D.Fretz s.n. (CAN [CAN556282]). **New York:** Nassau Co.:Jones Beach, 40°35'40"N, 73°30'10"W, 18 Aug 1932, H.A.Gleason & A.C.Smith 149 (CAN [CAN162198]). **Washington:** Jefferson Co.: mouth of Dosewallips River, E of Route 101, SW of Sylopash Point, 1 m, T25N R2W S2, 47°41.4'N, 122°53.5'W, 9 Sep 2004, P.F.Zika & F.Weinmann 20160 (WTU [WTU359724, Suppl. Fig. 30]). **Oregon:** Lane Co.: Cox Island, Siuslaw Estuary, 2.5 km E of Florence, 43.9716°N, 124.0672°W, 9 Aug 1983, R.E.Frenkel 3060 (UBC [UBCV196070, Suppl. Fig. 31]); Lane Co.: center of Cox Island in Siuslaw River estuary, 3.5 km E of Florence, SW corner Sec 30, T. 18S, R. 11 W., W.M., 43.9716°N, 124.0672°W, 22 Oct 1977, R.E.Frenkel s.n. (WTU [WTU286900, Suppl. Fig. 32]).

### Spartina pectinata

Link, Jahrb. Gewächsk. 1(3): 92-93. 1820. Type.

http://species-id.net/wiki/Spartina_pectinata

Spartina pectinata Link, Jahrb. Gewächsk. 1(3): 92-93. 1820. **Type**. United States of America:Aus Nordamerika, Bosc s.n. (holotype: B; isotype: US-92011! (fragm. ex B)).
Spartina cynosuroides fo. *major* St.-Yves, Candollea 5: 61, 62. 1932. **Type.** UNITED STATES OF AMERICA. Illinois: marais, Aug 1846, Riehl 488 (lectotype: P [P02243295!], here designated).
Spartina michauxiana Hitchc., Contr. U.S. Natl. Herb. 12(3): 153. 1908. *Spartinacynosuroides* var. *michauxiana* (Hitchc.) St.-Yves, Candollea 5: 58, t. 3, f. a-7. 1932. **Type.** United States of America. Illinois: hauteurs des terres, Michaux s.n. (holotype: P, not seen). Note: *Spartina michauxiana* is treated as a synonym of *Spartina pectinata* following Hitchcock (1951) and Mobberley (1956).
Spartina michauxiana var. *suttiei* Farw., Rep. Michigan Acad. Sci 21: 352.1920. *Spartina pectinata* var. *suttiei* (Farw.) Fernald, Rhodora 35: 260. 1933. **Type.** United States of America. Michigan: Island Lake, 16 Jul 1905, Farwell 1487a (holotype: probably BLH; isotype:GH [GH24445!]).
Spartina michauxiana var. *tenuior* Farw., Rep. Michigan Acad. Sci 21: 352.1920. **Type**. United States of America. Michigan: River Rouge, 15 Sep 1918, Farwell 5138 (holotype: probably BLH; isotype: MICH [MICH1108792!]).
Spartina pectinata fo. *variegata* Vict., Naturaliste Canad. 71: 209. 1944. **Type**. CANADA. Quebec: Ile Parrot (archipel d’Hochelaga’ sur le lac St-Louis), 12 Jul 1941, Victorin & Rolland 56603 (holotype: MT, not seen).

#### Description.

See Mobberley (1956), Barkworth (2003), and Douglas et al. (2001). 2*n* = 40 (Marchant 1963, 1968b, Reeder 1977).

#### Common name.

Prairie cordgrass.

#### Etymology.

The epithet *pectinata* means comb-like or tooth-like, and was given in reference to the distinctly pectinate teeth on the glume keels of *Spartina pectinata*, one of its diagnostic characteristics.

#### Illustrations.

Hitchcock 1935:490, Hitchcock 1951:510, Holmgren et al. 1998:749, Barkworth 2003:251, Cope and Gray 2009:553.

#### Distribution.

*Spartina pectinata* is widespread across much of North America north of Mexico, distributed in southern Alberta, eastern Washington and Oregon, south to Texas, and east to Newfoundland and Labrador (Barkworth 2003). It grows in marshes, slough and floodplains, in dry prairies and along roads (Barkworth 2003). It is known from a few scattered sites in Britain and Ireland where it has been introduced and escaped from cultivation (Cope and Gray 2009).

#### Comments.

*Spartina pectinata* is not considered to be a problematic invasive species, though it has been introduced sporadically to other regions, such as the United Kingdom. It occurs natively in eastern Washington and Oregon (Hitchcock et al. 1969, Barkworth 2003). *Spartina pectinata* is grown as an ornamental.

**Figure 10.**
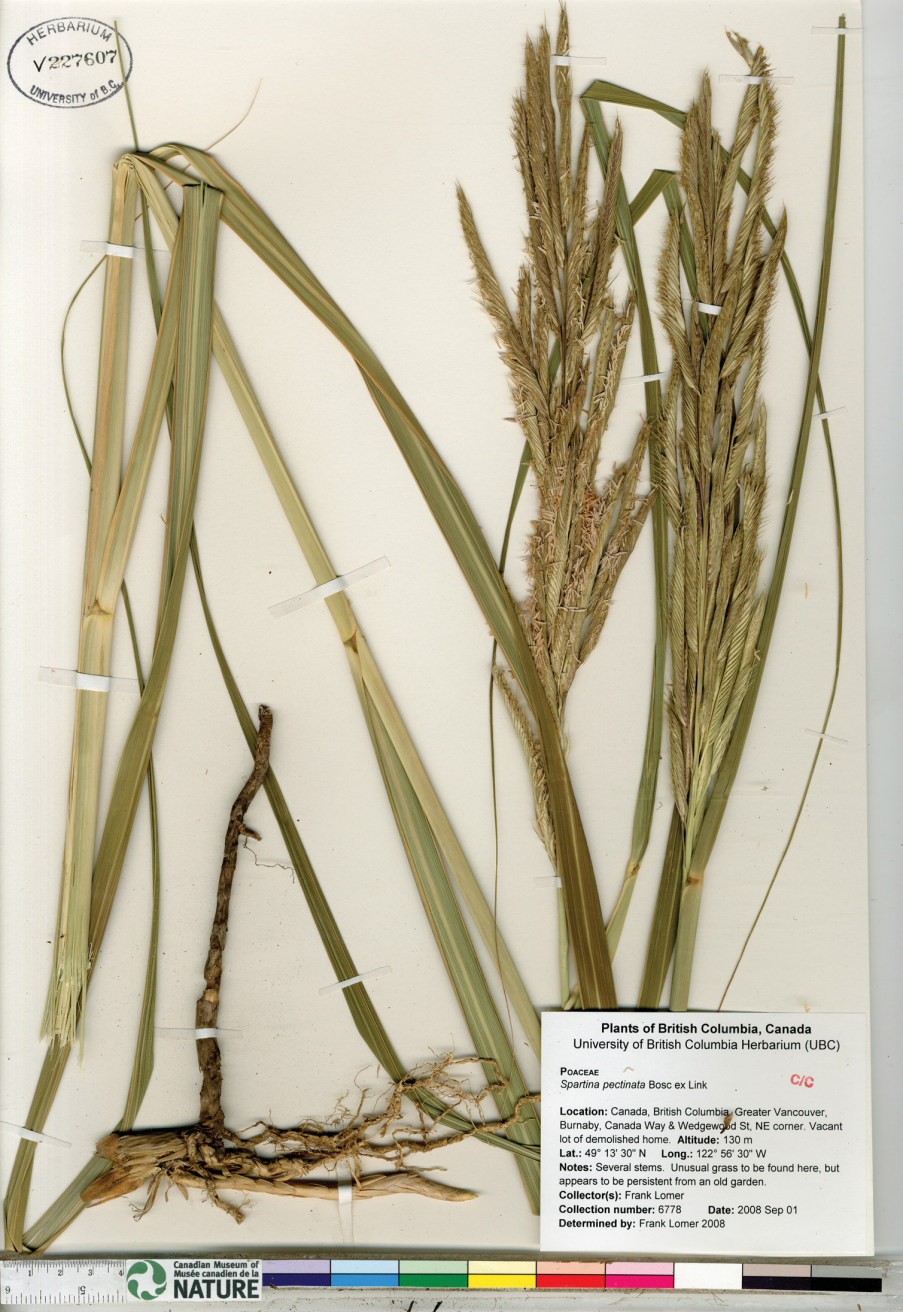
Photograph of a specimen of *Spartina pectinata* collected in Burnaby, British Columbia (Lomer 6778, UBC). Image published with the permission of the University of British Columbia Herbarium, Beaty Biodiversity Museum.

British Columbia is the only Canadian province in which *Spartina pectinata* is not native, but the taxon has been variously recognized as part of its flora. In the first major treatment of the British Columbia flora, Joseph K. Henry (1915) reported the non-native *Spartina cynosuroides*, an Atlantic coast taxon confused at the time with *Spartina pectinata* according to Eastham (1947), which Henry suggested might be present in the southern interior based on its presence in adjacent Washington. No collections of *Spartina pectinata* from the southern interior of British Columbia have been made. *Spartina pectinata* was added to the flora of British Columbia by Eastham (1947), based on a single collection made by V. Brink on the shore of Sea Island at the mouth of the Fraser River (where the Vancouver International Airport is now located). Eastham suggested that it might have been transported to this location down the river from the interior, but this is unlikely since there are no reports of the taxon from interior British Columbia. Based on this single record, *Spartina pectinata* was recognized as part of the British Columbia flora in subsequent floristic works (Hubbard 1969, Taylor and MacBryde 1977, Scoggan 1978), but was later excluded by Douglas et al. (1994) on the basis of being an introduced species not collected in over 40 years. This exclusion was followed in recent treatments (Qian and Klinka 1998, Douglas et al. 2001). Attempts to locate the Brink collection in British Columbia herbaria (V, UBC) were unsuccessful, and it is therefore not possible to confirm this report.. *Spartina pectinata* was recently collected in a vacant lot in the city of Burnaby (Lomer 6778, Fig. 10, 6805, Suppl. Fig. 33), where it likely escaped or persisted from cultivation (F. Lomer, personal communication, 2011). The clump from which these specimens were obtained no longer exists, as the site has been built over (F. Lomer, personal communication, 2012). The specimens reported here are the first confirmed records of *Spartina pectinata* for British Columbia. The species is not considered to be invasive.

#### Morphology.

*Spartina pectinata* is readily distinguished from all other taxa in the Pacific Northwest by its conspicuously awned glumes [vs. glumes unawned or short-awned (awns to 2 mm long), the latter state sometimes present in *Spartina gracilis*], and glume keels with robust, comb-like teeth [vs. glume keels that are glabrous, scabrous or ciliate]. Additional characters are given under other taxa.

#### Specimens examined.

**CANADA. British Columbia:** Greater Vancouver, Burnaby, Canada Way and Wedgewood St., NE corner, vacant lot of demolished home, 49°13'30"N, 122°56'30"W, 130 m, 01 Sep 2008, F.Lomer 6778 (UBC [UBCV227607, Fig. 10]); same location, 15 Sep 2008, F.Lomer 6805 (UBC [UBCV227406, Suppl. Fig. 33]). **Manitoba:** Rural Municipality of Pembina, NE of Darlingford and E of Manitou, 49°14'12"N, 98°18'04"W, 437 m, 16 Jul 2007, J.M.Saarela 1019 (CAN [CAN590577]). **United States of America.** **Colorado:** Fort Collins, 3 Aug 1898, n.c. 3551 (CAN [CAN162205]). **Idaho:** Oneida Co.: American Falls, 28 Jul 1911, A.Nelson & J.F.Macbride 1394 (CAN [CAN162207]). **Kansas**: S of Quinter, 16 Oct 1937, C.Brown s.n. (CAN [CAN162202]). **Maine:** Aroostook Co.: valley of the St. John River, Big Black River Rapids, Township xv, Range 13, 26 Jul 1917, H.St.John & G.E.Nichols 2135 (CAN [CAN162203]). **Minnesota:** Kittson Co.: Hallock, 16 Jul 1986, F.W.Schueler 16461 (CAN [CAN536117]). St. Louis Co.: beach of Esquagama Lake, 4 Aug 1944, O.Lakela 5647 (CAN [CAN162197]). **New Hamsphire:** Rockingham Co.: Newfields, along Squamscott River below bridge to Stratham, 8 Aug 1973, A.R.Hodgden & F.L.Steele 19838 (CAN [CAN555731]). Strafford Co.: Milton, edge of pond by railroad, 15 Jul 1959, A.R.Hodgden & F.L.Steele 11095 (CAN [CAN555851]). **New York:** Madison Co.: shore of Oneida Lake, South Bay, 27 Jun 1921, H.D.House 8289 (CAN [CAN162204]); St. Lawrence Co.: Morristown, 44°35'08"N, 75°38'53"W, 15 Aug 1914, O.P.Phelps 156 (CAN [CAN162209]). **North Dakota:** Lamoure Co.: along creek ½ mil W, 2 ¼ mi N of Edgeley, Nora Township, 46°21'34"N, 98°42'44"W, 24 Aug 1937, J.H.Moore & M.Moore 10094 (CAN [CAN198696]). **Ohio:** Lucas Co.: SE corner of Monclova Township, low bank of Maumee River at SW corner of Maumee city limits, 10 Sep 1967, R.L.Stuckey 5782 (CAN [CAN320720]). **Oregon:** Bars of Snake River, Ballard’s Landing, 8 Jul 1899, Wm.C.Cusick 2221 (CAN [CAN162206]). **South Dakota:** Aberdeen, Aug 1969, S.N.Stephenson s.n. (CAN [CANB432790]).

### Spartina ×townsendii

H. Groves & J. Groves, Bot. Exch. Club Rep. 1880. 37. 1881.

http://species-id.net/wiki/Spartina_×townsendii

Spartina ×townsendii H. Groves & J. Groves, Bot. Exch. Club Rep. 1880. 37. 1881. **Type.** England. Mud flats, near Hythe, South Hants, 1 Sep 1879, H.Groves s.n. (holotype: BM [BM001003965!]; isotypes: C, K [K000710272!], W [W19160030795!, W19160030798!] (ex hb. Groves), US [US1127161!] (fragm. ex W ex hb. Groves), US [US878793!]). Note: The location of the holotype has not been reported previously in the literature.
Spartina ×neyrautii Fouc., Ann. Soc. Sci. Nat. Charente-Maritime 31: 8. 1894. **Type.** France. près de Hendaye (Basses-Pyrénnées), E-J.Neyraut s.n., 24 Jul 1892 (isotypes: P [P00753804!, P03457326!, P03457334!, P03457416!], US! (fragm. ex P)]).

#### Description.

Culms 46–100 cm tall, thick, fleshy, rhizomatous. Sheaths glabrous; ligules 1–1.5 mm long; blades 6.5–37 cm long × 4–10 mm wide, flat proximally, often involute distally, divergent 30–40° from culms, adaxial surfaces glabrous, occasionally with very sparse hairs proximally, when present hairs to 0.2 mm long, abaxial surfaces glabrous, occasionally with sparse hairs proximally, when present hairs to 0.5 mm long, margins smooth. Inflorescences 10.5–24(–36) cm long × 7–25 mm wide at midpoint, erect, with (2)3–6(–10) branches; branches (6–)7.5–15(–18) cm long × (2.5–)3–4 mm wide, appressed or ascending, rachises 1–1.9 mm wide between spikelets, extending 2–10(–18) mm beyond the terminal spikelet, extension occasionally absent, glabrous, margins glabrous, occasionally with a few marginal hairs, when present hairs to 0.2 mm long. Spikelets 14–17.5 mm long × 1.5–2.5 mm wide, weakly appressed, weakly overlapping, calluses 0.6–1.5(–2) mm long. Glumes weakly to moderately pubescent, hairs 0.1–0.2 mm long, proximal hairs occasionally to 0.6 mm long, keels glabrous, ciliate or scabrous, when present hairs and teeth 0.2–0.5 mm long, usually longest proximally; lower glumes 7–13 mm long × 0.5–0.7 mm wide, 1-veined, tips acuminate or obtuse; upper glumes 12.5–16.5 mm long × 1–1.5 mm wide, 3-veined, tips acuminate or obtuse. Lemmas 9.5–13.5 mm long, 1-3–veined, pubescent distally, glabrous proximally, margins membranous, keels ciliate distally, hairs to 0.2 mm long, glabrous proximally. Paleas exceeding lemmas by ca. 1 mm, glabrous. Anthers 5–7(–8.5) mm long, not or incompletely exserted at maturity, indehiscent, medium to dark brown, pollen sterile; caryopses absent. 2*n* = 62 (Marchant 1963, 1968b).

#### Common name.

Townsend’s cordgrass.

#### Etymology.

The epithet *townsendii* was given in honour of the English botanist Frederick Townsend (1822–1905).

#### Illustration.

Cope and Gray 2009:547.

#### Distribution.

This species is found in England, Wales, Scotland, Ireland (Cope and Gray 2009), Italy (Scarton et al. 2003), United States (Washington), New Zealand (Partridge 1987), and Canada (British Columbia).

#### Comments.

In the mid to late 1800s an unknown cordgrass of restricted distribution appeared and spread rapidly along the shores of Southampton Water, England (Stapf 1914), which differed morphologically (particularly by its sterile pollen) from *Spartina maritima*, the single cordgrass species native to the Atlantic coast of Europe and north Africa (Marchant and Goodman 1969c), and the introduced *Spartina alterniflora*, which had been present in the region since the early part of the 19^th^ century. The brothers Henry and James Grove (1881) described this taxon as *Spartina townsendii* from plants collected near Hythe. In the 1890s a second form of *Spartina townsendii*, which was recognized and considered distinct by having fertile stamens, was collected at multiple localities in the region, and by the mid twentieth century it had expanded substantially on tidal flats across the British Isles (see Goodman et al. 1959, Hubbard 1957, 1965). For decades these two forms (one sterile, the other fertile) of *Spartina townsendii* were referred to collectively as the *Spartina townsendii* aggregate or *Spartina townsendii* sensu lato. Because of its vigorous growth and ability to rapidly colonize and stabilize mud flats, *Spartina townsendii* s.l. was considered to be a “useful” species and was distributed and planted widely for land reclamation, coastal protection, and animal feed across the British Isles, Europe, and in New Zealand (e.g., Oliver 1925, Harboard 1949, Goodman et al. 1959, Ranwell 1967).

Soon after its formal description *Spartina townsendii* was considered to be a species of hybrid origin. Foucaud (1894) suggested that *Spartina townsendii* was probably a hybrid of the native *Spartina maritima* and the introduced *Spartina alterniflora*, a hypothesis later supported by Stapf (1914) and Huskins (1930), who examined cytological evidence and hypothesized that fertile plants of *Spartina townsendii* s.l. originated from chromosome doubling following hybridization between its parent species. Marchant (1963) confirmed this work, and reported chromosome numbers as 2*n* = 62 for *Spartina alterniflora*, 2*n* = 60 for *Spartina maritima*, 2*n* = 62 for sterile plants of *Spartina townsendii*, and 2*n* = 120, 122, 124 for fertile plants of *Spartina townsendii*. The hybrid and chromosome doubling origins of the forms of *Spartina townsendii* have been confirmed by multiple lines of molecular evidence (Guénégou et al. 1988, Raybould et al. 1991, Ferris et al. 1997, Ayres and Strong 2001, Baumel et al. 2003). Hubbard (1957) observed the type specimen of *Spartina townsendii* to be the sterile F1 hybrid, and the taxon was subsequently referred to as *Spartina ×townsendii*. The fertile plants remained without a name until Hubbard (1978) later described them as *Spartina anglica*, and the two forms have since been recognized as distinct taxa. Molecular data have identified *Spartina alterniflora* as the female parent and *Spartina maritima* as the male parent in the origin of *Spartina ×townsendii* (e.g., Ferris et al. 1997).

An independent origin of *Spartina ×townsendii* is documented in France. In 1894 Jules Foucaud described *Spartina neyrautii* Fouc. from southwestern France and northern Spain. *Spartina neyrautii* was initially considered to be a variant of *Spartina maritima* (e.g., Chevalier 1923, Saint-Yves 1932), but was later recognized as a morphologically and cytologically distinct hybrid, *Spartina ×neyrautii*,with the same parentage as *Spartina ×townsendii* (e.g., Jovet 1941, Chevalier 1933, Marchant 1977). Baumel et al. (2003) confirmed this with molecular data, demonstrating that *Spartina ×neyrautii* and *Spartina ×townsendii* originated independently by hybridization between the same maternal (*Spartina alterniflora*) and paternal (*Spartina maritima*) taxa. Because both taxa apply to the hybrid *Spartina alterniflora × S. maritima*, the later name *Spartina ×neyrautii* is a synonym of *Spartina ×townsendii*, in accordance with article H.2 of the Vienna Code (McNeill et al. 2006), and as noted earlier by Raybould et al. (1990). In recent decades it has been documented that these hybrid plants are highly restricted in distribution in France (Marchant 1977, Hubbard et al. 1978, Raybould et al. 1990, Baumel et al. 2003). Minor morphological differences between *Spartina ×neyrautii* and *Spartina ×townsendii* were noted by Marchant (1977). Measurements of spikelet characters in *Spartina ×neyrautii* type material examined at US fall within the range of variation reported here for *Spartina ×townsendii*.

*Spartina ×townsendii* has been introduced into North America, where apparently only a single occurrence has been reported in the literature. Hitchcock et al. (1969) noted a single known population of *Spartina ×townsendii* in Washington at Stanwood, Snohomish Co. At the time of that publication, the fertile (=*Spartina anglica*) and non-fertile forms of *Spartina ×townsendii* were not distinguished taxonomically, and it is not explicitly clear from the flora which form of the taxon was known from the site. A specimen collected in 1965 from this population [Austenson s.n. (WTU, Suppl. Fig. 35)] is here confirmed to be the F1 sterile hybrid *Spartina ×townsendii*. The determination of a more recent collection from the Stanwood area identified as *Spartina ×townsendii* requires confirmation (Snohomish Co., Davis Slough west of Stanwood, 25 Aug 1990, M.Arnot 254, WTU-317391, not seen). A 2005 collection from Washington originally identified as *Spartina ×townsendii* (Giblin & Legler 270 WTU) is here re-determined to be *Spartina anglica*. Barkworth (2003) included *Spartina ×townsendii* in her treatment of *Spartina* for North America, but did not include a distribution map or otherwise indicate a range for the species, suggesting some confusion in the literature on its status in North America. Kozloff (2005) also included *Spartina ×townsendii* (as well as *Spartina anglica*) in his Pacific Northwest flora, indicating only ‘coastal salt marshes’ for its distribution. There apparently are no published data on the current status of the Stanwood population. If the population at Stanwood persists, new collections should be made to document its continued existence at the site, and if other populations are known or discovered, herbarium collections should be made to document their existences.

**Figure 11.**
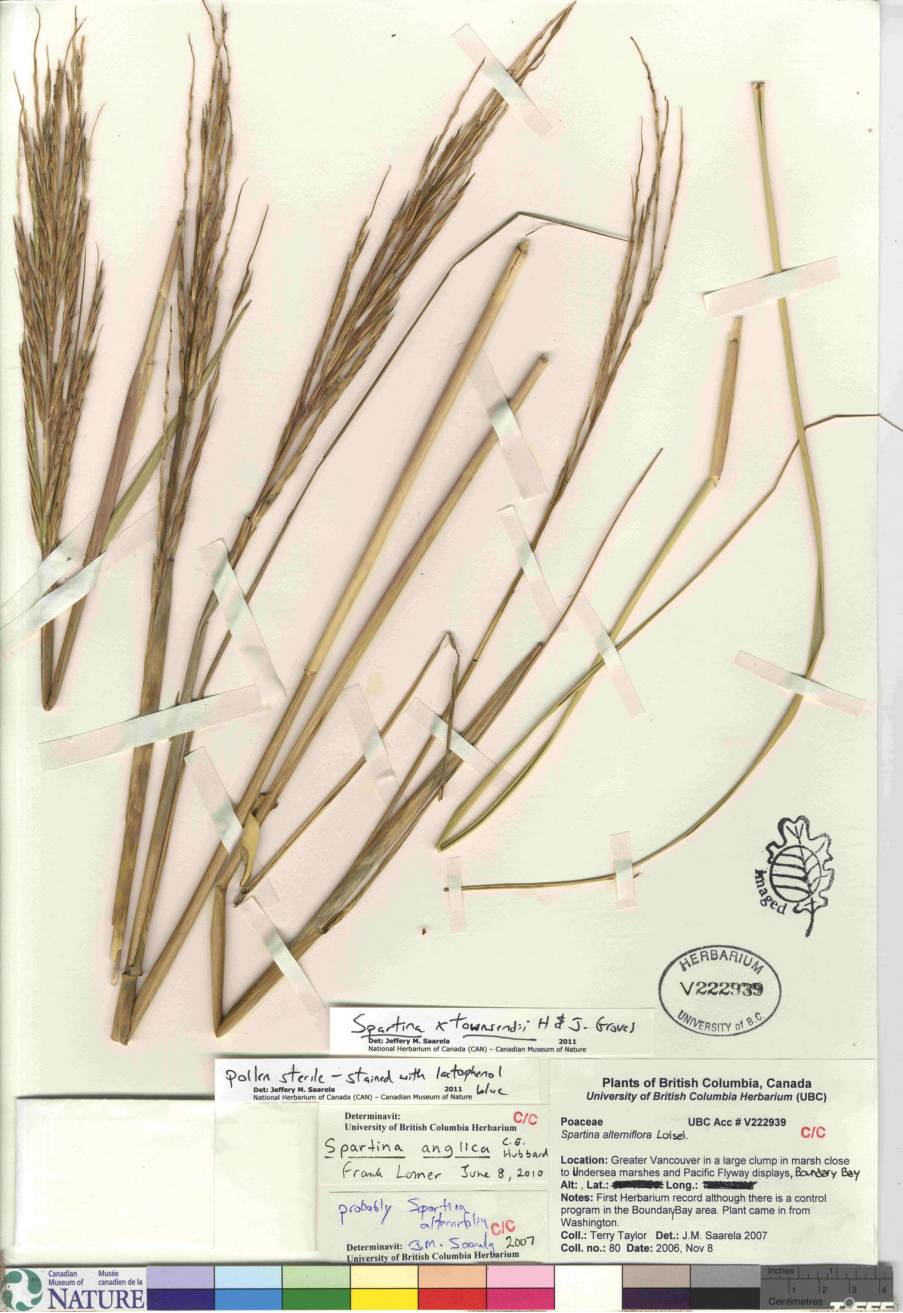
Photograph of a specimen of *Spartina ×townsendii* collected in Boundary Bay, British Columbia (Taylor 80, UBC). This is the first record of the taxon for British Columbia. Image published with the permission of the University of British Columbia Herbarium, Beaty Biodiversity Museum.

**Figure 12.**
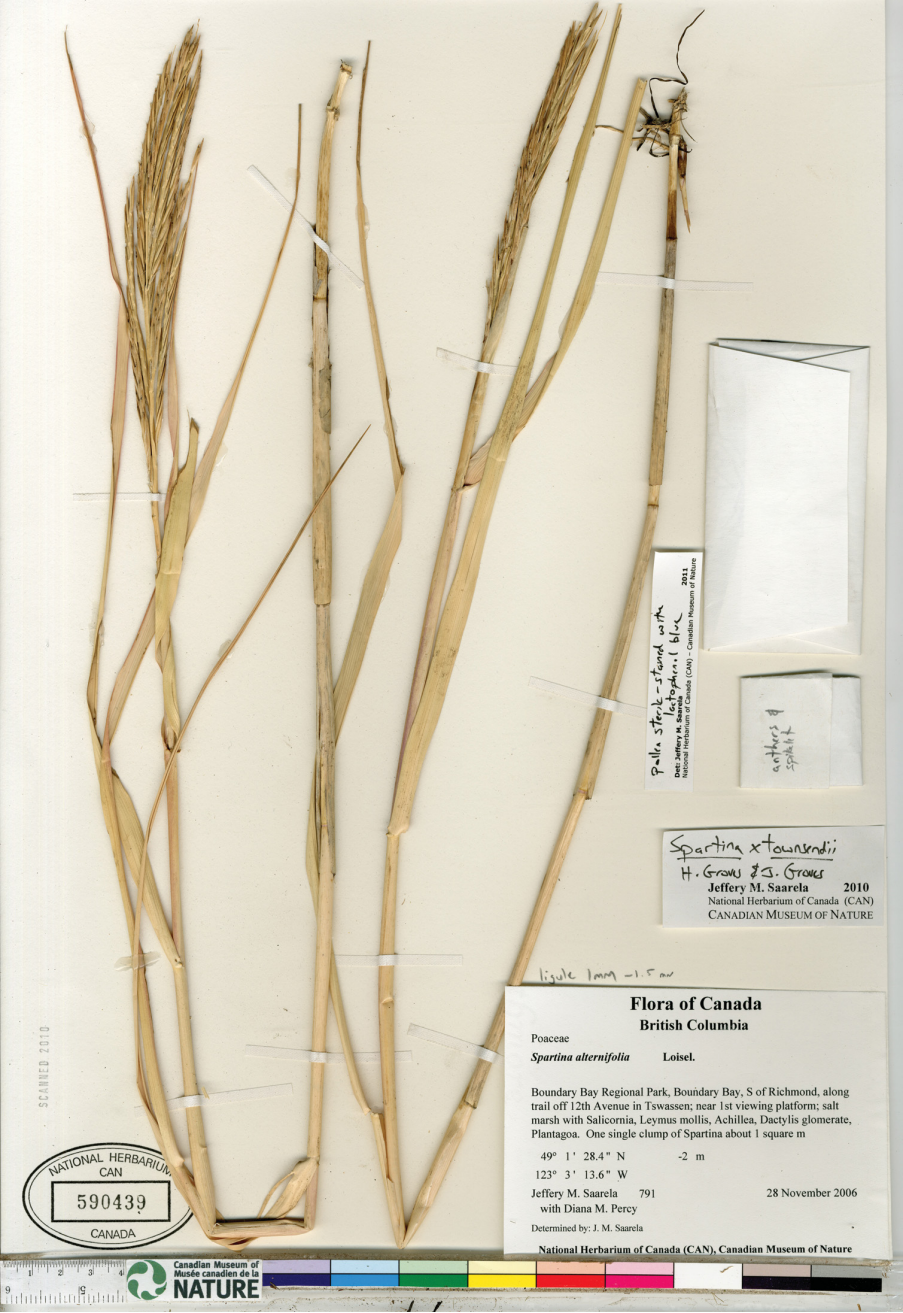
Photograph of a specimen of *Spartina ×townsendii* collected in Boundary Bay, British Columbia (Saarela and Percy 791, CAN). This is the second record of the taxon for British Columbia.

*Spartina ×townsendii* has not previously been reported from British Columbia. It is here reported as new for the province on the basis of two collections made in 2006 in in Boundary Bay at sites separated by some 4.4 kilometers (by air)[Taylor 80 (UBC, Fig. 11) and Saarela & Percy 791 (CAN, Fig. 12, UBC, Suppl. Fig. 34)]. These appear to be the most recent confirmed reports of the taxon in North America since it was collected at Stanwood, Washington. Herbarium specimens of these collections were initially determined (incorrectly) as *Spartina anglica* and *Spartina alterniflora*, since *Spartina ×townsendii* was not expected in British Columbia. Subsequent study of this material, in combination with the *Spartina* taxonomic literature and comparisons with specimens of *Spartina anglica* and Old World specimens of *Spartina ×townsendii* at CAN and UBC, confirmed the specimens to be *Spartina ×townsendii*, prompting the current taxonomic study. Pollen in these specimens is sterile, as determined by pollen staining (see discussion under *Spartina anglica*, Fig. 5), further confirming their identities as *Spartina ×townsendii*. Specimens from which pollen was extracted and stained with lactophenol cotton blue to assess fertility are identified with the symbol † in the Specimens Examined below.

The origin of *Spartina ×townsendii* in British Columbia is not known, and there are no data on the extent of the Boundary Bay sub-populations in 2006 aside from notes on the Saarela and Percy collection label indicating a single clump of the grass approximately one meter in diameter. It is not known if *Spartina ×townsendii*has persisted in British Columbia since collected some five years ago. Since major efforts are ongoing to remove *Spartina* plants from Boundary Bay where *Spartina ×townsendii*was found, it is possible the original stands from which the specimens were collected have been removed. The region should be studied to determine if the taxon is present. Since the taxon is sterile and does not set seed, it must have been introduced into Boundary Bay by vegetative reproduction, probably from rhizome fragments transported in tidal currents. It is possible that the British Columbia plants originated from the stand at Stanwood, Washington, if it persists, or there may be other extant occurrences of *Spartina ×townsendii* somewhereto the south of Boundary Bay. Workers searching Puget Sound for invasive *Spartina* (e.g., Benbrook 2011) should be aware that *Spartina ×townsendii* may also be present.

#### Morphology.

The description here is based on the first known collection from Washington, the two collections from British Columbia, and Old World material housed at CAN and UBC (see Specimens Examined), including collections made by H. & J. Groves who first described the taxon over a century ago. The North American specimens of *Spartina ×townsendii* are morphologically similar to the Old World specimens examined. *Spartina ×townsendii* is distinguished from *Spartina anglica* by its shorter spikelets [(14–17.5 mm long vs. (15–)16.5–25 mm long]; shorter anthers [5–7(–8.5) mm long vs. 7–10 mm long]; indehiscent anthers that are not or incompletely exserted with sterile pollen [vs. dehiscent anthers that are usually fully exserted with fertile pollen; see Figs 4, 5]; shorter ligules [1–1.5 mm long vs. 1–3 mm long]; upper glumes 3-veined [vs. upper glumes 3–6-veined]; and shorter upper glumes [12.5–16.5 mm long vs. 13–22 mm long]. The angle of the leaf blade with the stem is 30–40° in *Spartina ×townsendii*, compared to 30–60° in *Spartina anglica* (Marchant 1968a). This character can be difficult to evaluate on herbarium specimens depending on how they were pressed, but should be more reliable in the field for distinguishing the taxa, particularly if they occur together. If stands of *Spartina ×townsendii* are relocated in British Columbia or elsewhere in the region, the taxonomic utility of this character should be carefully evaluated. Marchant (1968a) noted swards of *Spartina ×townsendii* to be distinct in appearance from swards of *Spartina anglica* in England, having high culm density and high tiller density (ca. 96/100 cm^2 ^vs. ca. 52/100 cm^2^). It is not clear how *Spartina ×townsendii* differs morphologically from *Spartina alterniflora × S. foliosa* hybrids that have been documented in California (Daehler and Strong 1997, Ayres et al. 1999, 2003, 2004, Anttila et al. 2000).

#### Specimens examined.

**Canada. British Columbia: Greater Vancouver Regional District:** Boundary Bay Regional Park, Boundary Bay, S of Richmond along trail off 12 Avenue in Tsawwassen, near 1^st^ viewing platform, 49°01'28"N, 123°03'14"W, ca. 0 m, 28 Nov 2006, J.M.Saarela & D.M.Percy 791 (CAN [CAN590439†, Fig. 12, UBC [UBCV228476†, Suppl. Fig. 34]); Greater Vancouver in marsh close to Undersea marshes and Pacific Flyway displays, Boundary Bay, 49°03'34"N, 123°01'27"W [secondary], 8 Nov 2006, T.Taylor 80 (UBC [UBCV222939†, Fig. 11). **United States of America. Washington:** Snohomish Co.:near Stanwood, ca. 48°14'N, 122°21'W, 26 Aug 1965, H.M.Austenson s.n. (WTU [WTU229915†, Suppl. Fig. 35]). **England.** Hampshire Co.: Hythe, South Hants, 9 Oct 1883, H.Groves s.n. (US [US555778]); Hayling Island, 13 Sep 1900, E.S.Marshall s.n. (CAN [CAN585633, Suppl. Fig. 36]); Southampton, 50°53'49"N, 01°24'15"W, Sep 1904, H.Groves & J.Groves 4596 (CAN [CAN251679†, Suppl. Fig. 37], US [US1535531]); Lymington, Keyhaven, 50°47'N, 00°58'W, 28 Aug 1977, G.Halliday 457/77 (CAN [CAN522593†, Suppl. Fig. 38]); Keyhaven, 50°43'22"N, 01°34'10"W, 30 Jul 1966, G.Halliday 100/66 (CAN [CAN301583, Suppl. Fig. 39]); Hants, Aug 1877, J.Groves s.n. (CAN [CAN421009†, Suppl. Fig. 40]); Hythe, Southampton, central marshes, male sterile, 50.8667°N, 01.3999°W uncertainty 7193 m, 10 Sep 1959, C.Marchant s.n. (UBC [UBCV221074, Suppl. Fig. 41]); N Hayling Island, Duckard Point, male sterile, 50.8051°N, 0.9778°W uncertainty 7194 m, 4 Aug 1960, C.Marchant s.n. (UBC [UBCV221101, Suppl. Fig. 42]); N Hayling Island, male sterile, 50.8051°N, 0.9778°W uncertainty 7194 m, 17 Aug 1961, C.Marchant s.n. (UBC [UBCV221098, Suppl. Fig. 43]); Eling, male sterile, 50.8999°N, 1.4833°W uncertainty 7193 m, 29 Aug 1961, C.Marchant s.n. (UBC [UBCV221100, Suppl. Fig. 44, UBC221095, Suppl. Fig. 45]); Hythe, south marshes, male sterile, 50.8666°N, 1.3999°W uncertainty 7193 m, 28 Oct 1959, C.Marchant s.n. (UBC [UBCV221093, Suppl. Fig. 46, UBCV221099, Suppl. Fig. 47]); Hythe, central marsh, near Sylvan Villa, giant male sterile, 50.8666°N, 1.3999°W uncertainty 7193 m, 16 Aug 1961, C.Marchant s.n. (UBC [UBCV221097, Suppl. Fig. 48, UBCV221096, Suppl. Fig. 49]); Hythe, Hants, male sterile, 50.8666°N, 01.3999°W uncertainty 7193 m, 10 Sep 1959, C.Marchant s.n. (UBC [UBCV221094, Suppl. Fig. 50]).

## Supplementary Material

XML Treatment for Spartina

XML Treatment for Spartina alterniflora

XML Treatment for Spartina anglica

XML Treatment for Spartina densiflora

XML Treatment for Spartina gracilis

XML Treatment for Spartina patens

XML Treatment for Spartina pectinata

XML Treatment for Spartina ×townsendii

## Appendix B

**Supplementary figures**

Exemplar *Spartina* herbarium specimens. (doi: 10.3897/phytokeys.10.2734.app) File format: JPG/ZIP.

**Explanation note:** Photographs of herbarium specimens of *Spartina alterniflora*, *Spartina anglica*, *Spartina densiflora*, *Spartina gracilis*, *Spartina patens*, *Spartina pectinata*, and *Spartina ×townsendii*. These are a subset of the specimens examined in the current study, as cited in the main text.

**Citation:** Saarela JM (2012) Taxonomic synopsis of invasive and native *Spartina* (Poaceae, Chloridoideae) in the Pacific Northwest (British Columbia, Washington and Oregon), including the first report of *Spartina ×townsendii* for British Columbia, Canada. PhytoKeys 10: 25–82. doi: 10.3897/phytokeys.10.2734.app

Supplementary figure 1. *Spartina alterniflora*, Maxwell 1575 (WTU342828). Image published with the permission of University of Washington Herbarium, Burke Museum.

Supplementary figure 2. *Spartina alterniflora*, Lebovitz s.n. (WTU344373). Image published with the permission of University of Washington Herbarium, Burke Museum.

Supplementary figure 3. *Spartina alterniflora*, Zika 18935 (WTU371783). Image published with the permission of University of Washington Herbarium, Burke Museum.

Supplementary figure 4. *Spartina anglica*, Williams 2004-3 (UBCV220132). Image published with the permission of the University of British Columbia Herbarium, Beaty Biodiversity Museum.

Supplementary figure 5. *Spartina anglica*, Williams 2004-2 (CAN592131).

Supplementary figure 7. *Spartina anglica*, Walker 382 (WTU373426). Image published with the permission of University of Washington Herbarium, Burke Museum.

Supplementary figure 8. *Spartina anglica*, Zika 17595 (WTU365225). Image published with the permission of University of Washington Herbarium, Burke Museum.

Supplementary figure 9. *Spartina anglica*, R.E. Frenkel 3045 (UBCV196071). Image published with the permission of the University of British Columbia Herbarium, Beaty Biodiversity Museum.

Supplementary figure 10. *Spartina anglica*, Frenkel 3045 (WTU305390). Image published with the permission of University of Washington Herbarium, Burke Museum.

Supplementary figure 11. *Spartina anglica*, Weinmann 233 (WTU370619). Image published with the permission of University of Washington Herbarium, Burke Museum.

Supplementary figure 12. *Spartina anglica*, Giblin 244 (WTU364297). Image published with the permission of University of Washington Herbarium, Burke Museum.

Supplementary figure 13. *Spartina anglica*, Giblin 270 (WTU364317). Image published with the permission of University of Washington Herbarium, Burke Museum.

Supplementary figure 14. *Spartina anglica*, Zika 19170 (WTU355108). Image published with the permission of University of Washington Herbarium, Burke Museum.

Supplementary figure 15. *Spartina anglica*, Jørgensen & Svendsen s.n. (CAN358861).

Supplementary figure 16. *Spartina anglica*, Matthews s.n. (CAN448159).

Supplementary figure 17. *Spartina anglica*, Riddelsjell s.n. (CAN467908).

Supplementary figure 18. *Spartina anglica*, Thompson s.n. (UBCV1679). Image published with the permission of the University of British Columbia Herbarium, Beaty Biodiversity Museum.

Supplementary figure 19. *Spartina anglica*, Taylor 5378 (UBCV20934). Image published with the permission of the University of British Columbia Herbarium, Beaty Biodiversity Museum.

Supplementary figure 20. *Spartina anglica*, Taylor 5379 (UBCV69243). Image published with the permission of the University of British Columbia Herbarium, Beaty Biodiversity Museum.

Supplementary figure 21. *Spartina anglica*, Melvill 1841 (UBCV1678). Image published with the permission of the University of British Columbia Herbarium, Beaty Biodiversity Museum.

Supplementary figure 22. *Spartina anglica*, Oliver s.n. (CAN421006).

Supplementary figure 23. *Spartina densiflora*, Lomer 7377 (CAN).

Supplementary figure 24. *Spartina densiflora*, Heimer 01-01 (UBCV224048). Image published with the permission of the University of British Columbia Herbarium, Beaty Biodiversity Museum.

Supplementary figure 25. *Spartina densiflora*, Heimer 01-01 (WTU349303). Image published with the permission of University of Washington Herbarium, Burke Museum.

Supplementary figure 26. *Spartina gracilis*, Macoun 77227 (CAN33944).

Supplementary figure 27. *Spartina gracilis*, Macoun s.n. (CAN33943).

Supplementary figure 28. *Spartina gracilis*, Kujala & Cajander s.n. (CAN394014).

Supplementary figure 29. *Spartina patens*, Lomer 87-001 (UBCV194265). Image published with the permission of the University of British Columbia Herbarium, Beaty Biodiversity Museum.

Supplementary figure 30. *Spartina patens*, Zika 20160 (WTU359724). Image published with the permission of University of Washington Herbarium, Burke Museum.

Supplementary figure 31. *Spartina patens*, Frenkel 3060 (UBCV196070). Image published with the permission of the University of British Columbia Herbarium, Beaty Biodiversity Museum.

Supplementary figure 32. *Spartina patens*, Frenkel s.n. (WTU286900). Image published with the permission of University of Washington Herbarium, Burke Museum.

Supplementary figure 33. *Spartina pectinata*, Lomer 6805 (UBCV227406). Image published with the permission of the University of British Columbia Herbarium, Beaty Biodiversity Museum.

Supplementary figure 34. *Spartina ×townsendii*, Saarela & Percy 791 (UBCV228476). Image published with the permission of the University of British Columbia Herbarium, Beaty Biodiversity Museum.

Supplementary figure 35. *Spartina ×townsendii*, Austenson s.n. (WTU229915). Image published with the permission of University of Washington Herbarium, Burke Museum.

Supplementary figure 36. *Spartina ×townsendii*, Marshall s.n. (CAN585633).

Supplementary figure 37. *Spartina ×townsendii*, Groves & Groves 4596 (CAN251679).

Supplementary figure 38. *Spartina ×townsendii*, Halliday 457/77 (CAN522593).

Supplementary figure 39. *Spartina ×townsendii*, Halliday 100/66 (CAN301583).

Supplementary figure 40. *Spartina ×townsendii*, Groves s.n. (CAN421009).

Supplementary figure 41. *Spartina ×townsendii*, Marchant s.n. (UBCV221074). Image published with the permission of the University of British Columbia Herbarium, Beaty Biodiversity Museum.

Supplementary figure 42. *Spartina ×townsendii*, Marchant s.n. (UBCV221101). Image published with the permission of the University of British Columbia Herbarium, Beaty Biodiversity Museum.

Supplementary figure 43. *Spartina ×townsendii*, Marchant s.n. (UBCV221098). Image published with the permission of the University of British Columbia Herbarium, Beaty Biodiversity Museum.

Supplementary figure 44. *Spartina ×townsendii*, Marchant s.n. (UBCV221100). Image published with the permission of the University of British Columbia Herbarium, Beaty Biodiversity Museum.

Supplementary figure 45. *Spartina ×townsendii*, Marchant s.n. (UBCV221095) Image published with the permission of the University of British Columbia Herbarium, Beaty Biodiversity Museum.

Supplementary figure 46. *Spartina ×townsendii*, Marchant s.n. (UBCV221093). Image published with the permission of the University of British Columbia Herbarium, Beaty Biodiversity Museum.

Supplementary figure 47. *Spartina ×townsendii*, Marchant s.n. (UBCV221099). Image published with the permission of the University of British Columbia Herbarium, Beaty Biodiversity Museum.

Supplementary figure 48. *Spartina ×townsendii*, Marchant s.n. (UBCV221097). Image published with the permission of the University of British Columbia Herbarium, Beaty Biodiversity Museum.

Supplementary figure 49. *Spartina ×townsendii*, Marchant s.n. (UBCV221096). Image published with the permission of the University of British Columbia Herbarium, Beaty Biodiversity Museum.

Supplementary figure 50. *Spartina ×townsendii*, Marchant s.n. (UBCV221094). Image published with the permission of the University of British Columbia Herbarium, Beaty Biodiversity Museum.
